# Short-term photovoltaic power forecasting using DTW-based K-Medoids clustering and a hybrid VMD-CEEMDAN-1DCNN-S-Mamba model

**DOI:** 10.1038/s41598-026-57372-x

**Published:** 2026-06-19

**Authors:** Hu Tianxiang, Gao Li, ChenLi Tianxi, Xie Congjiu, Xu Mingshen, Wu Peng

**Affiliations:** 1 Department of Mathematics and Physics, North China Electric Power University, Baoding, 071000 China; 2 Department of Electrical Engineering, North China Electric Power University, Baoding, 071000 China; 3 Department of Automation, North China Electric Power University, Baoding, 071000 China; 4 Kaituo Navigation Control Technology Co., Ltd., Beijing, 100000 China; 5 Department of Mathematics and Physics, North China Electric Power University, Baoding, 071000 China; 6 Engineering Training and Innovation Entrepreneurship Center, North China Electric Power University, Baoding, 071000 China

**Keywords:** Energy science and technology, Engineering, Mathematics and computing

## Abstract

The short-term prediction of photovoltaic (PV) power is critical for grid stability and efficient renewable energy dispatch. However, the strong volatility of PV generation due to weather changes poses major challenges for accurate forecasting. This paper proposes a hybrid model combining DTW-based K-Medoids clustering, two-stage VMD-CEEMDAN decomposition, and a 1DCNN-S-Mamba network for multi-step PV power forecasting.Historical PV power and irradiance sequences are clustered into sunny, cloudy, and rainy patterns using K-Medoids with Dynamic Time Warping (DTW), which is more robust to outliers and temporal shifts than traditional K-Means. Variational Mode Decomposition (VMD) extracts the main trend components, while Complete Ensemble Empirical Mode Decomposition with Adaptive Noise (CEEMDAN) is applied to the residual to further mitigate high-frequency noise. The decomposed components, along with meteorological variables and weather-type codes, are input into the 1DCNN-S-Mamba model, where the 1DCNN captures local multiscale features and the bidirectional S-Mamba models long-range dependencies.Tested on a two-year dataset with a 5-min resolution from three Australian PV stations, the model achieves an $R^2$ of 0.9822, an MAE of 0.1188 kW, and an RMSE of 0.2451 kW. It outperforms eleven benchmark models, especially under cloudy and rainy conditions. Ablation studies confirm the effectiveness of the two-stage decomposition and the 1DCNN module. The proposed framework provides a practical solution for high-accuracy PV power forecasting.

## Introduction

The increased global energy consumption indicates that fossil fuels can no longer meet the demands of sustainable development, highlighting the paramount role of renewable energy sources^1,2^. Among these, solar power is highly advantageous due to its environmental friendliness, vast abundance, and long-term sustainability, making it a crucial pillar of future energy systems^3,4^. However, photovoltaic (PV) power generation is inherently intermittent and highly sensitive to weather conditions, which poses significant challenges to grid stability and consistent power supply. Consequently, highly accurate short-term PV power predictions are urgently needed^5–8^. These predictions facilitate real-time power balancing, effective generator scheduling, and informed decision-making, thereby helping to develop an intelligent, AI-powered future energy ecosystem^9^.

Photovoltaic power prediction can be broadly divided into two main categories: physical models and data-driven models. Physical models rely on meteorological variables and the electrical characteristics of PV cells to calculate power generation^10,11^. While theoretically rigorous, they require exceptionally high-quality meteorological data, are highly computationally intensive, and struggle to accurately simulate rapid weather transitions or local shading effects^12^. Conversely, data-driven techniques have gained widespread popularity due to their strong nonlinear fitting capabilities and efficient utilization of historical data. Among these, time-series deep learning models have emerged as the state-of-the-art algorithms for PV power forecasting^13–16^.

Early data-driven approaches primarily relied on traditional machine learning methods, which were later superseded by deep learning models like Recurrent Neural Networks (RNN)^17^, LSTM^18^, and CNN^19^. In particular, LSTM has been extensively utilized^20–22^ for its ability to mitigate the vanishing gradient problem. To further reduce computational costs, the Gated Recurrent Unit (GRU) was adopted as a streamlined alternative^23–25^. Meanwhile, Temporal Convolutional Networks (TCN) have been introduced to extract multi-scale local features, significantly reducing predictive error rates^26^. Nonetheless, these conventional recurrent and convolutional architectures fundamentally struggle to simultaneously model ultra-long sequence dependencies and abrupt behavioral shifts during extreme weather conditions^27,28^.

To overcome the limitations in modeling global receptive fields, Transformer-based models have recently emerged as the new frontier^29^. Powered by the self-attention mechanism, advanced variants such as Informer^30^ and Autoformer^31^ effectively capture long-range global meteorological trends while reducing quadratic computational complexity^32^. Despite their superior performance, Transformer-based models typically require massive datasets for convergence and suffer from high memory consumption and significant computational overhead during training. This necessitates the exploration of more lightweight and efficient sequence modeling paradigms.

To more accurately capture multiscale spatiotemporal characteristics, recent studies have extensively explored hybrid models that combine signal decomposition algorithms with deep learning networks^33–36^. For instance, Complete Ensemble Empirical Mode Decomposition with Adaptive Noise (CEEMDAN) and Variational Mode Decomposition (VMD) have been employed to mitigate sequence non-stationarity^37–40^. Representative works, such as TCN-BiLSTM-Attention^41^ and WNPS-LSTM-Informer^42^, have shown great promise. However, existing hybrid approaches still face three significant limitations: (1) single-stage decomposition methods cannot always effectively filter out complicated high-frequency noise; (2) recurrent networks and Transformers incur excessively high time and resource costs when processing long sequences; and (3) inadequate feature fusion restricts the ability to integrate local variations with global dependencies.

To mitigate these identified shortcomings, a novel short-term PV power forecasting framework integrating Dynamic Time Warping (DTW)-based K-Medoids clustering, a two-stage VMD-CEEMDAN decomposition technique, and a 1DCNN-S-Mamba architecture is proposed in this paper. The framework operates through a systematic workflow: it begins with weather-type clustering, proceeds to a primary and secondary signal decomposition, and concludes with an integrated network that extracts local features while modeling long-range dependencies. By adopting this comprehensive pipeline, the proposed model effectively resolves key issues inherent in current methods, including the failure to capture high-frequency details, insensitivity to abrupt meteorological changes, and inefficiencies in long-sequence modeling.

Specifically, historical data is first categorized into three typical weather conditions (sunny, cloudy, and rainy) via DTW-based K-Medoids. Next, the VMD-CEEMDAN mechanism is applied to the PV sequence of each weather cluster. VMD extracts the main band-limited components, while CEEMDAN further decomposes the residuals to isolate high-frequency details, ensuring no critical minor signals are lost. Ultimately, the multivariate IMFs are processed by the integrated network. The 1DCNN refines local multiscale features, and the bidirectional S-Mamba efficiently captures long-range relationships with linear time complexity. This makes S-Mamba exceptionally well-suited for modeling the long-memory properties of PV power generation compared to conventional BiGRUs or Transformers.

The main contributions of this paper are summarized as follows:

- A novel weather-aware clustering strategy is introduced using the DTW-based K-Medoids algorithm. By utilizing actual data points as medoids and a shape-sensitive distance metric, this approach demonstrates superior robustness against outliers and time distortions compared to classical K-Means, thereby establishing a solid foundation for weather-specific forecasting.
- A two-stage VMD-CEEMDAN decomposition framework is proposed to comprehensively address the non-stationarity of PV power sequences. By employing CEEMDAN to further process VMD residuals, this mechanism successfully recovers crucial high-frequency micro-features that are typically lost in conventional single-stage decompositions, resulting in cleaner and more physically meaningful subcomponents.
- An innovative, lightweight 1DCNN-S-Mamba prediction architecture is designed. For the first time, it synergistically integrates the local multiscale feature extraction capability of dilated 1D convolutions with the global long-range dependency modeling of the bidirectional S-Mamba state-space model. Crucially, it achieves this with linear time complexity, offering significant computational advantages over traditional Transformer or BiLSTM-based designs.
- Extensive empirical validations and benchmark comparisons are conducted using a two-year, 5-minute resolution real-world dataset from three Australian PV plants. Comprehensive ablation studies and comparisons against eleven baseline models (including LSTM, GRU, TCN-BiLSTM-Attention, and standalone Mamba) confirm that the proposed framework achieves state-of-the-art predictive performance, especially under highly volatile and challenging rainy or cloudy conditions.

## Results

### Overview of the proposed framework

**Fig. 1:**
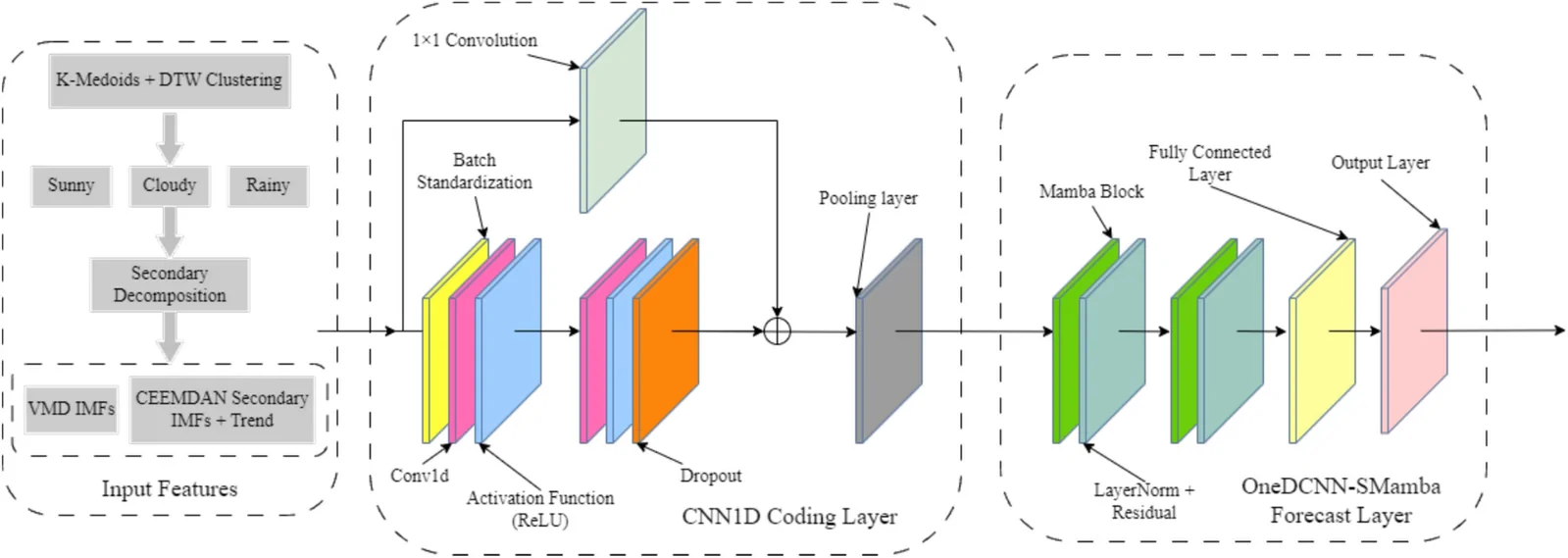
Architecture of the proposed 1DCNN-S-Mamba model.

As illustrated in Fig. 1, this study proposes a novel hybrid forecasting framework that integrates DTW-K-Medoids clustering, a two-stage VMD-CEEMDAN signal processing strategy, and a 1DCNN-S-Mamba network.

The workflow proceeds as follows: First, to mitigate the impact of weather volatility, the preprocessed photovoltaic data is clustered into three distinct weather profiles (sunny, cloudy, and rainy) via the unsupervised DTW-K-Medoids algorithm, allowing for condition-specific modeling. Subsequently, to handle the non-stationary nature of the power sequences, Variational Mode Decomposition (VMD) is applied. The highly nonlinear residual sequence generated from VMD is further processed by CEEMDAN to thoroughly extract complex local oscillations. Ultimately, all the extracted Intrinsic Mode Functions (IMFs), concatenated with the original exogenous meteorological features, are fed into the 1DCNN-S-Mamba model to capture spatiotemporal dependencies and output the final predictions. (Detailed mathematical formulations and parameter configurations for each module are provided in the Methods section at the end of this paper.)

#### Signal decomposition results

To effectively mitigate the non-stationarity and strong volatility inherent in photovoltaic (PV) power generation sequences, a two-stage VMD-CEEMDAN decomposition strategy was implemented. As the primary stage, VMD is utilized to extract the main periodic trends. However, the performance of VMD heavily relies on the pre-determined number of decomposition modes (*K*). To scientifically determine the optimal *K*, the center frequency observation method was rigorously applied to the highly volatile “Cloudy” cluster sequence to ensure algorithmic robustness.

**Table 1:** Center frequencies and minimum adjacent distances of VMD under different *K* values.

Number of modes (*K*)	Center frequencies of IMFs	Min adjacent distance
2	0.0022, 0.1321	0.1299
3	0.0022, 0.1320, 0.3086	0.1297
4	0.0022, 0.1216, 0.1530, 0.3094	0.0315
**5**	**0.0022, 0.1214, 0.1526, 0.3045, 0.3710**	**0.0312**
6	0.0006, 0.0064, 0.1241, 0.1565, 0.3049, 0.3713	0.0057
7	0.0006, 0.0063, 0.1241, 0.1563, 0.3044, 0.3693, 0.4785	0.0057
8	0.0006, 0.0062, 0.1217, 0.1504, 0.2229, 0.3075, 0.3707, 0.4787	0.0056

Significant values are in bold.

As detailed in Table 1, when *K* increases from 2 to 5, the center frequencies of the extracted Intrinsic Mode Functions (IMFs) are well-spaced, indicating distinct dynamic features are effectively separated. However, a critical turning point occurs at *K=6*, where the minimum distance between adjacent frequencies drops abruptly to 0.0057, forcibly generating two highly similar low-frequency modes (0.0006 and 0.0064). This phenomenon signifies severe over-decomposition and mode aliasing. Consequently, *K=5* was selected as the optimal parameter to balance feature extraction sufficiency and mode orthogonality.

**Fig. 2:**
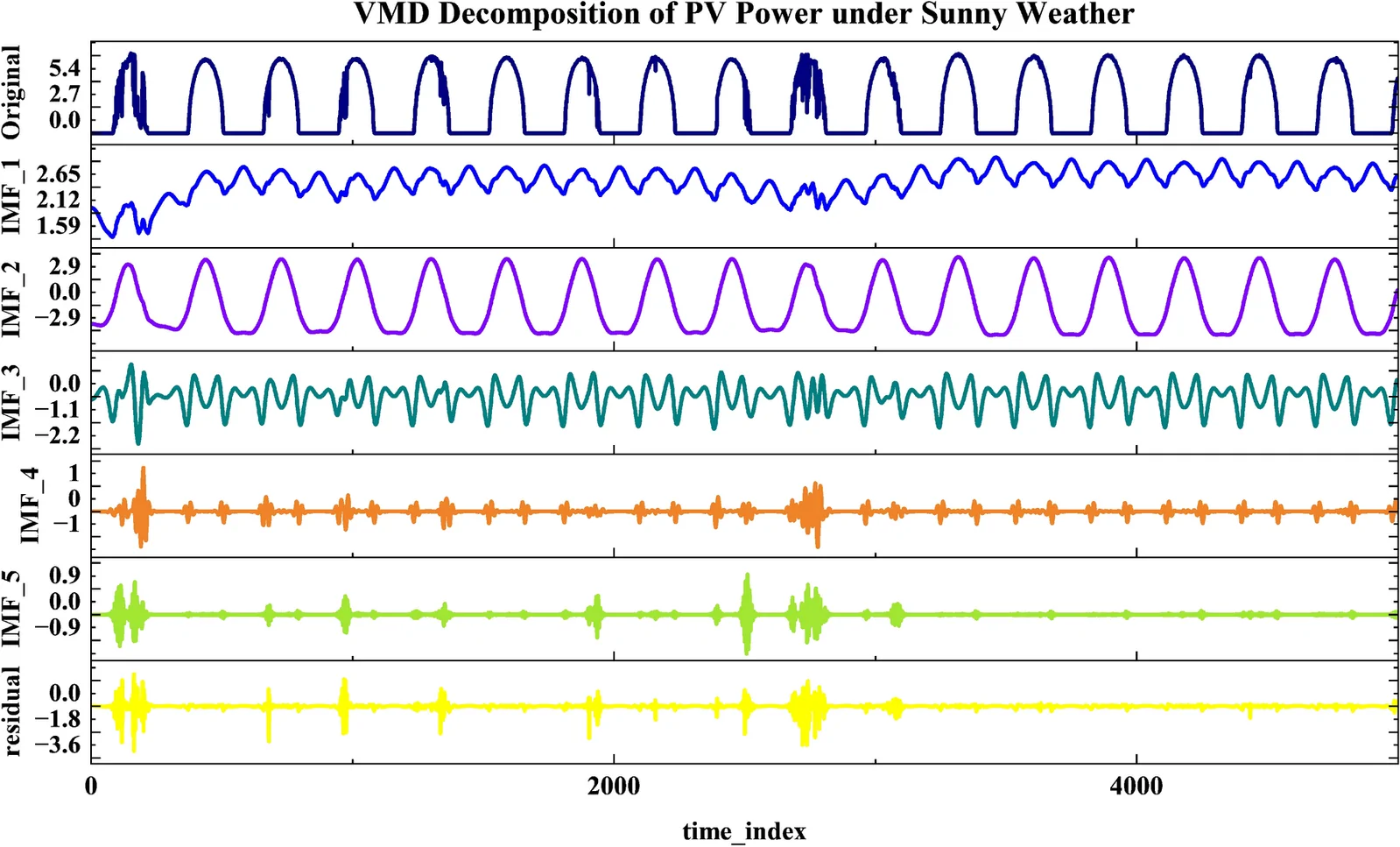
VMD decomposition results for the Sunny weather type.

Based on this optimal setting, Fig. 2 illustrates the first-stage VMD results, using the “Sunny” weather cluster as a representative example. The original PV power signal is adaptively decomposed into five IMFs (IMF1–IMF5) and a residual component across different frequency bands. Notably, IMF2 captures the dominant and highly regular diurnal cycle of solar generation, while the other IMFs represent low-frequency trends and high-frequency fluctuations.

However, despite the efficiency of VMD in isolating the main frequency components, a complex residual sequence remains, as shown in Fig. 2. This residual still exhibits prominent sharp spikes and high-frequency non-linear fluctuations (potentially caused by transient meteorological anomalies or sensor noise), which are difficult for a single VMD algorithm to fully resolve. If directly fed into the prediction model, these highly volatile residual features could degrade forecasting accuracy.

**Fig. 3:**
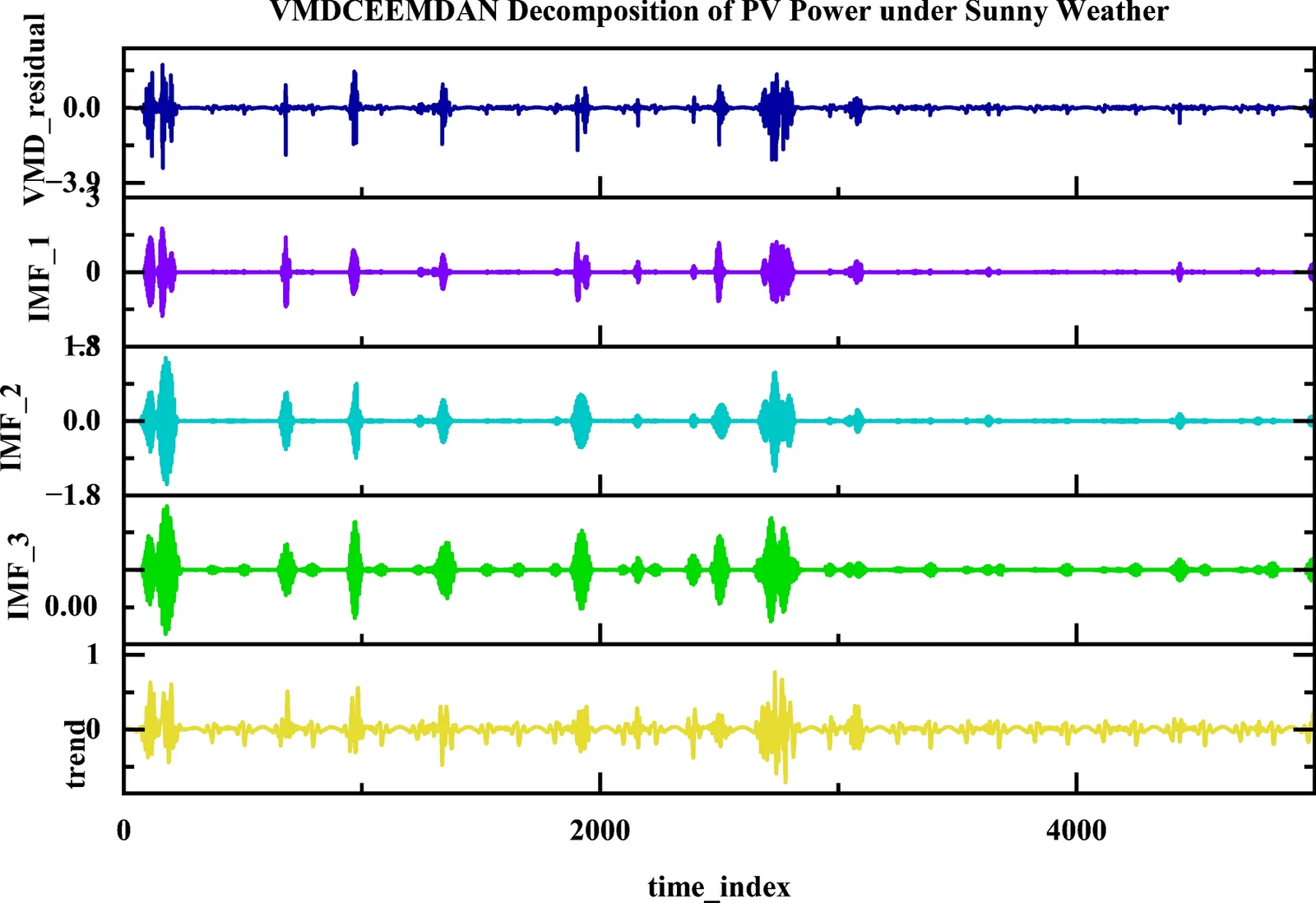
CEEMDAN decomposition results for the Sunny weather type.

To address this limitation and fully mine the hidden temporal patterns, CEEMDAN was introduced as the secondary decomposition stage specifically targeting the VMD residual. As depicted in Fig. 3, the complex VMD residual is further decomposed into three more stable sub-components (IMF1–IMF3) and a final trend. This secondary process successfully isolates the high-frequency random bursts into distinct, stable oscillatory modes. Consequently, through this VMD-CEEMDAN strategy, the originally chaotic PV signal is effectively transformed into a series of stationary and predictable sub-sequences, which significantly reduces the learning burden for the subsequent 1DCNN-S-Mamba network.

The primary advantage of this hybrid two-stage decomposition framework lies in its complementary nature: VMD reliably extracts stable, low-frequency principal modes, while CEEMDAN adeptly refines the high-frequency volatility and noise embedded within the residual. Consequently, this approach not only improves the overall predictability and interpretability of the data but also provides an optimal, highly structured input for the subsequent predictive model.

### Comparison experiments

#### Evaluation criteria

To comprehensively assess the predictive accuracy and robustness of the proposed framework, three standard statistical metrics were selected: Mean Absolute Error (MAE), Root Mean Square Error (RMSE), and the Coefficient of Determination ($R^2$). Specifically, MAE reflects the average magnitude of forecasting errors, RMSE imposes a larger penalty on significant deviations, and $R^2$ indicates the overall goodness-of-fit of the model. Their mathematical definitions are expressed as follows:

1 $$\begin{aligned} R^2 = 1 - \frac{\sum _{i=1}^{n} (y_i - \hat{y}_i)^2}{\sum _{i=1}^{n} (y_i - \bar{y})^2} \end{aligned}$$

2 $$\begin{aligned} \text {MAE} = \frac{1}{n} \sum _{i=1}^{n} |y_i - \hat{y}_i| \end{aligned}$$

3 $$\begin{aligned} \text {RMSE} = \sqrt{\frac{1}{n} \sum _{i=1}^{n} (y_i - \hat{y}_i)^2} \end{aligned}$$

where: $y_i$ is the actual power at step *i*. $\hat{y}_i$ is the predicted power at step *i*. *n* is the total number of samples in the test set

#### Comparison with baseline models

To comprehensively evaluate the validity and superiority of the proposed 1DCNN-S-Mamba model, extensive comparative experiments were conducted under three representative weather scenarios (sunny, cloudy, and rainy). All models evaluated in this section were trained and tested exclusively using the dataset from Station A to maintain data consistency.

To provide a rigorous and multi-dimensional benchmark, eleven representative and state-of-the-art baseline models were selected for comparison. These baselines can be categorized as follows: (1) Classic recurrent and convolutional networks: LSTM, GRU, BiLSTM, and TCN; (2) Hybrid deep learning architectures: CNN-LSTM, TCN-BiLSTM-Attention, and RF-KAN-GRU; (3) Advanced Transformer-based time series models: Informer, iTransformer, and TimeCMA; and (4) The standalone State Space Model: Mamba.

**Fig. 4:**
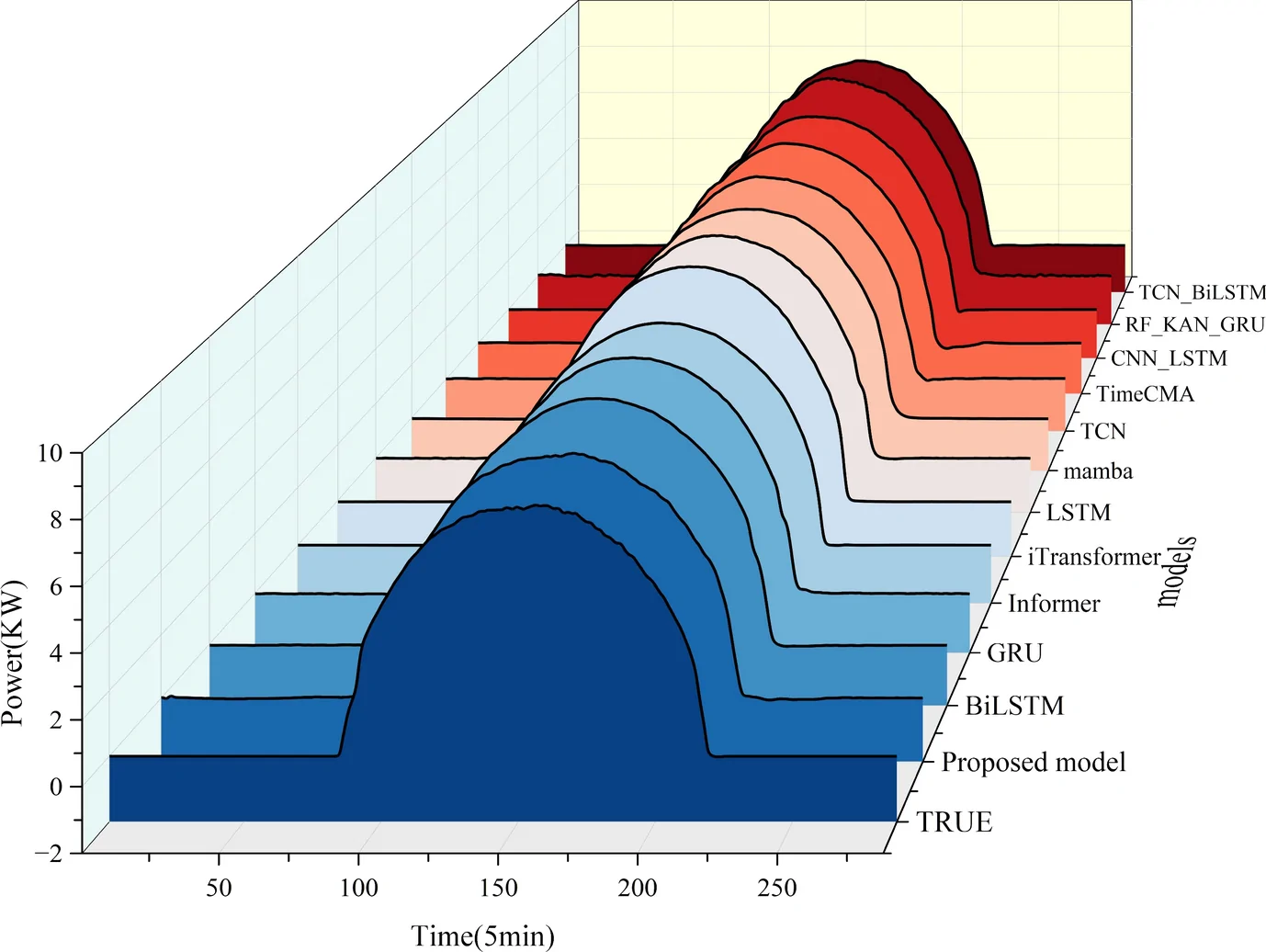
Model prediction curves under the Sunny weather conditions.

**Fig. 5:**
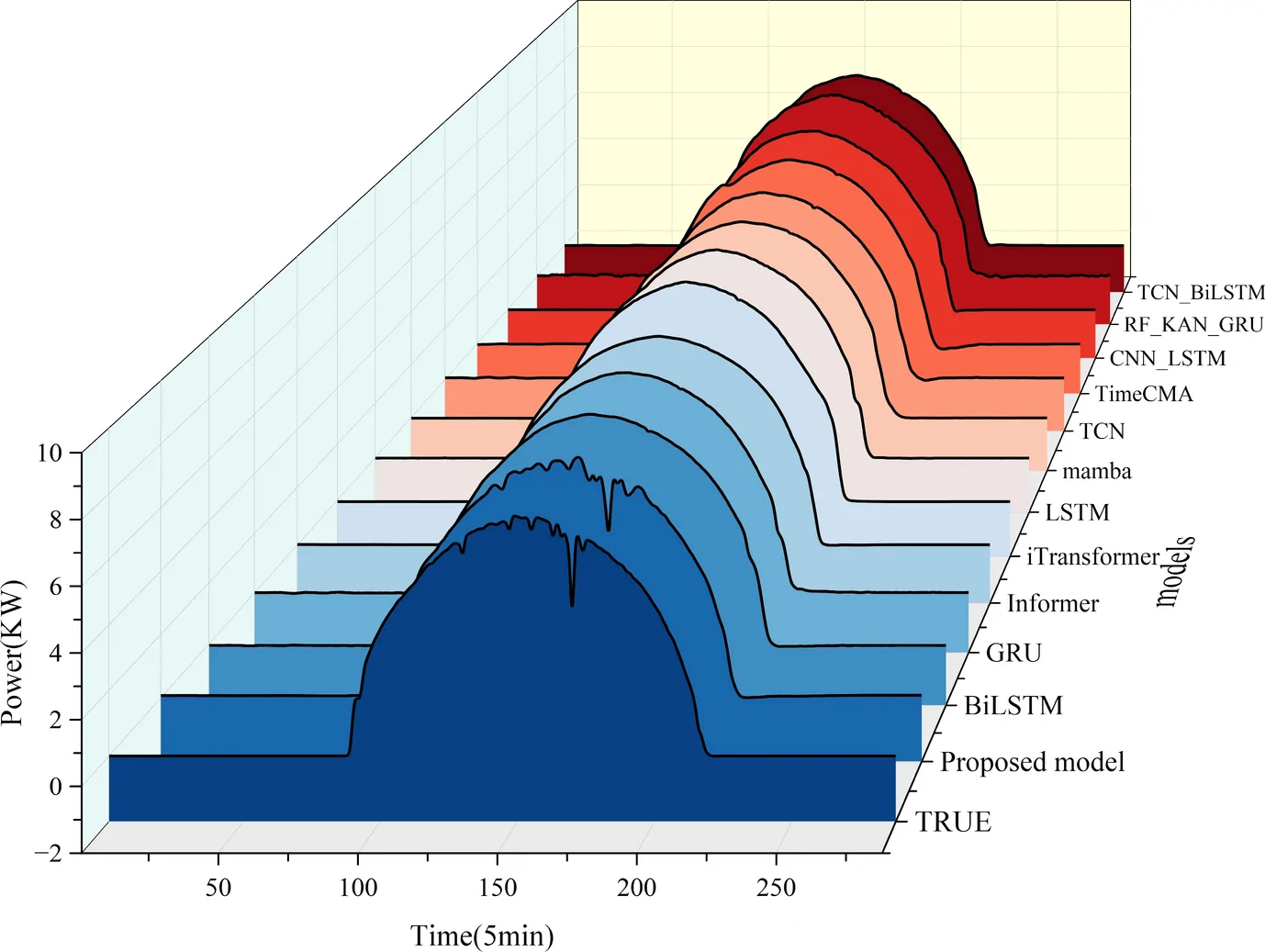
Model prediction curves under the Cloudy weather conditions.

**Fig. 6:**
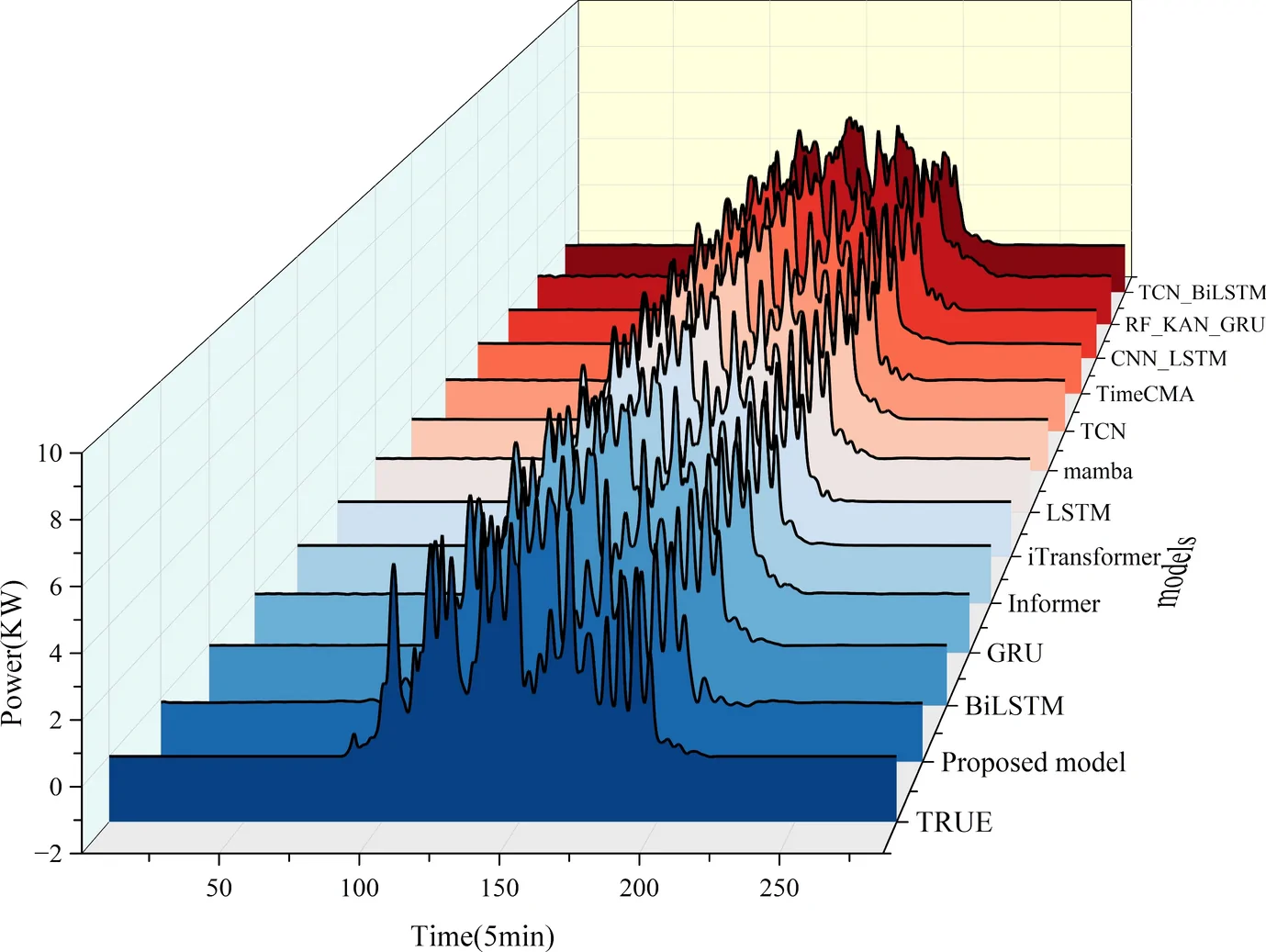
Model prediction curves under the Rainy weather conditions.

Crucially, to ensure strict fairness in the comparison, all the aforementioned baseline models were fed with the exact same input data sequences. Furthermore, they were trained using the identical training parameters and configurations (e.g., learning rate, batch size, epochs, and optimization algorithm) as those specified for the proposed 1DCNN-S-Mamba model. The intuitive forecasting trajectories of different models under the three distinct weather conditions are illustrated in Figs. 4, 5, and 6, respectively.

**Fig. 7:**
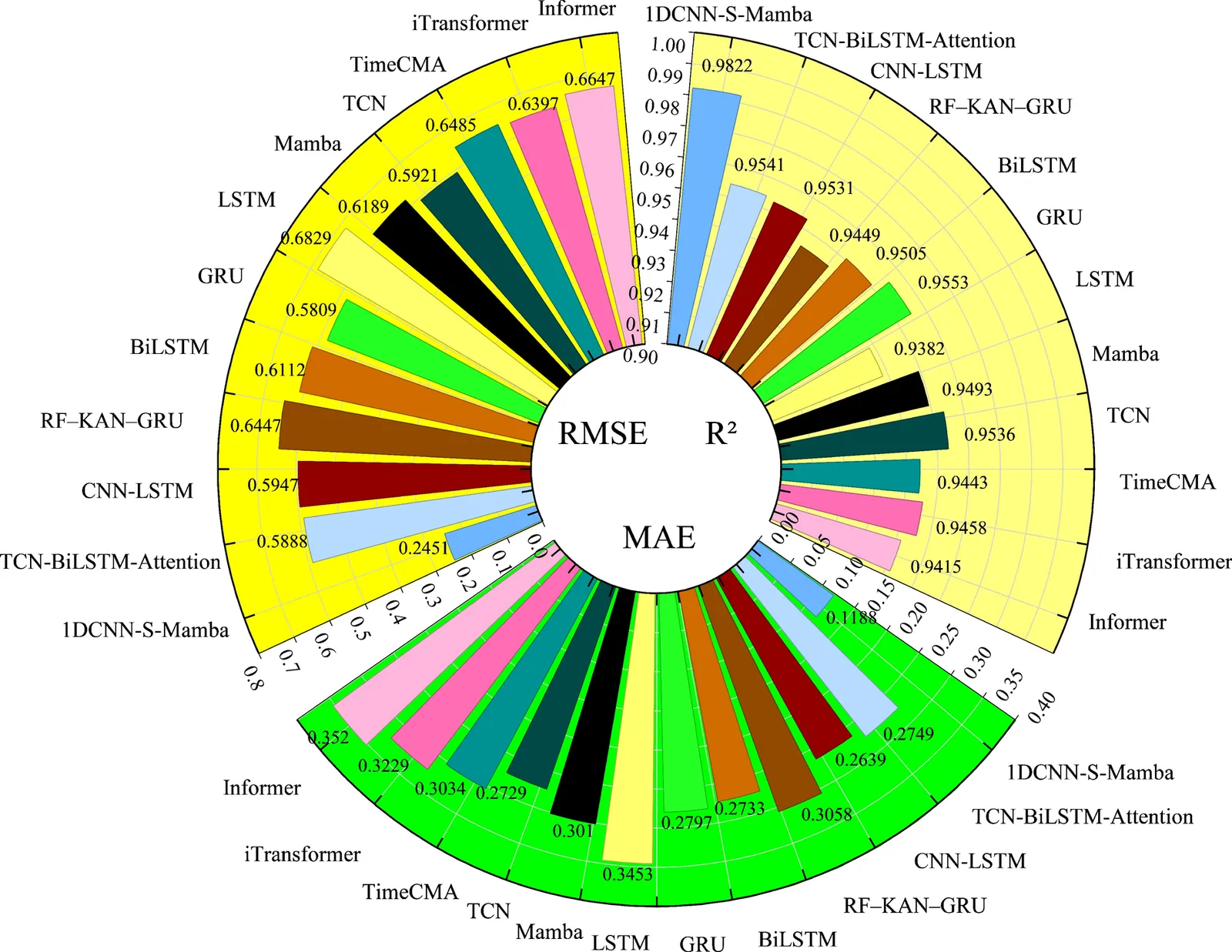
Comparison of evaluation metrics for different models.

The evaluation metrics of different models are compared in Fig. 7.

The quantitative results demonstrate the effectiveness of the proposed framework (i.e., DTW-K-Medoids-VMD-CEEMDAN-1DCNN-S-Mamba) in time series forecasting tasks. The overall outcomes of the comparative experiments validate that the proposed model significantly outperforms the diverse benchmark baselines across all evaluation metrics, confirming its state-of-the-art predictive capabilities. In terms of the coefficient of determination ($R^2$), the proposed model achieves a remarkable value of 0.9822, vastly surpassing all competitors. The most competitive baselines, GRU ($R^2 = 0.9553$) and TCN-BiLSTM-Attention ($R^2 = 0.9541$), lag behind the proposed model by absolute margins of 0.0269 and 0.0281, respectively. Statistically, this indicates that our framework can successfully explain 98.22% of the variance in the target data, exhibiting excellent goodness-of-fit and generalization capabilities.

**Fig. 8:**
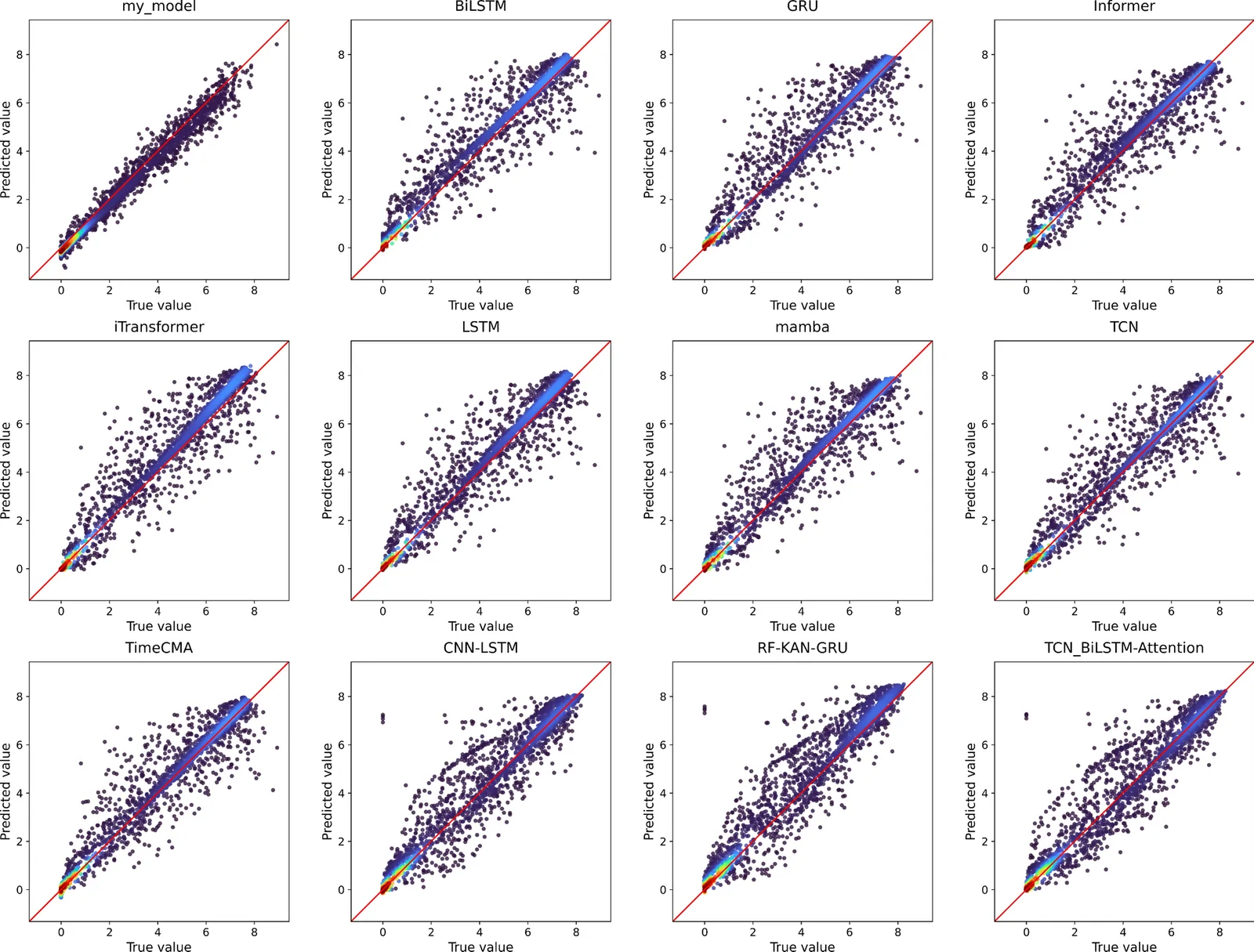
Prediction versus observation density scatter plots of different models.

To intuitively visualize this superior fitting performance, Fig. 8 presents the prediction versus observation density scatter plots for all evaluated models. As depicted, the scatter points of the proposed model are densely and symmetrically clustered along the ideal *y=x* reference line (solid red line), with high-density regions closely tracking the diagonal. This indicates minimal prediction bias and an extremely high correlation between the predicted and actual values. In contrast, the predictions from the baseline models exhibit varying degrees of wider dispersion and severe deviations from the reference line, particularly in the high-value regions (e.g., Informer, CNN-LSTM, and RF-KAN-GRU show significant underestimations and scatter). This powerful visual evidence perfectly aligns with the quantitative $R^2$ results, demonstrating clear dominance over classic recurrent neural networks (LSTM, GRU, BiLSTM), temporal convolutional networks (TCN), Transformer-based architectures (Informer, iTransformer), and the standalone Mamba model.

Regarding the specific error metrics, the advantages of the proposed model are even more pronounced:

1. Mean Absolute Error (MAE): The proposed model achieves the lowest MAE of 0.1188. This represents a substantial reduction of approximately 56.8% compared to the second-best model, TCN-BiLSTM-Attention (0.2749), and a 65.6% decrease relative to the standard LSTM (0.3453). This minimal average deviation between the predicted and actual values highlights the exceptional precision of the proposed method.
2. Mean Squared Error (MSE): Similarly, the MSE drops significantly to 0.0629, establishing a massive performance advantage. This constitutes an 81.4% reduction compared to the GRU (0.3375) and an 83.6% reduction compared to the standalone Mamba (0.3831). Since MSE penalizes larger errors more heavily, this steep decline confirms that the proposed model is highly effective at mitigating extreme prediction errors and exhibits strong robustness against outliers and complex data fluctuations.
3. Root Mean Square Error (RMSE): The RMSE of the proposed model is 0.2451, which is 58.4% lower than that of the TCN-BiLSTM-Attention (0.5888) and 64.1% lower than the LSTM. This substantial decrease in RMSE further verifies the reliability and stability of the framework for real-world engineering deployments.

Fundamentally, while standalone deep learning models (e.g., LSTM, GRU, TCN, or Mamba) exhibit basic forecasting capabilities, their significant performance gaps compared to our framework highlight the inherent limitations of single-architecture designs when handling complex, non-stationary, and multi-scale time series data. In contrast, the synergistic integration of DTW-K-Medoids clustering, VMD-CEEMDAN two-stage decomposition, and the 1DCNN-S-Mamba predictor organically unifies signal decomposition, local feature extraction, and global sequence modeling. This comprehensive pipeline ensures a much higher efficiency in capturing multi-frequency characteristics and long-range dependencies, ultimately driving the superior predictive performance.

### Ablation experiment

To rigorously validate the individual effectiveness and synergistic benefits of the core components within the proposed framework, a comprehensive ablation study was conducted. Specifically, the evaluation focused on the contributions of the DTW-based K-Medoids clustering, the two-stage VMD-CEEMDAN decomposition, and the spatial feature extraction of the 1DCNN module.

To ensure a strictly fair and meaningful assessment of each module’s contribution, all ablation variants were trained and evaluated under identical experimental conditions. This included the exact same dataset partitioning (an 8:2 chronological train-test split, with 15% of the training data allocated for validation), identical training hyperparameters, and consistent evaluation metrics.

The ablation experiments are primarily designed from the following two perspectives:

- Decomposition Strategy: Evaluating the necessity and performance gains provided by the VMD and CEEMDAN modules.
- Model Architecture: Isolating and verifying the specific contribution of the 1DCNN module within the 1DCNN-S-Mamba predictor.

The specific experimental configurations for all ablation variants are detailed in Table 2.

**Table 2:** Design of ablation experiments.

Decomposition strategy	Model architecture	Note
No VMD	CEEMDAN-1DCNN-S-Mamba	CEEMDAN decomposition only
No CEEMDAN	VMD-1DCNN-S-Mamba	VMD decomposition only
VMD+CEEMDAN	Proposed model	Complete model
VMD+CEEMDAN	VMD-CEEMDAN-S-Mamba	Remove the CNN1D module

#### Results of the ablation experiment

**Fig. 9:**
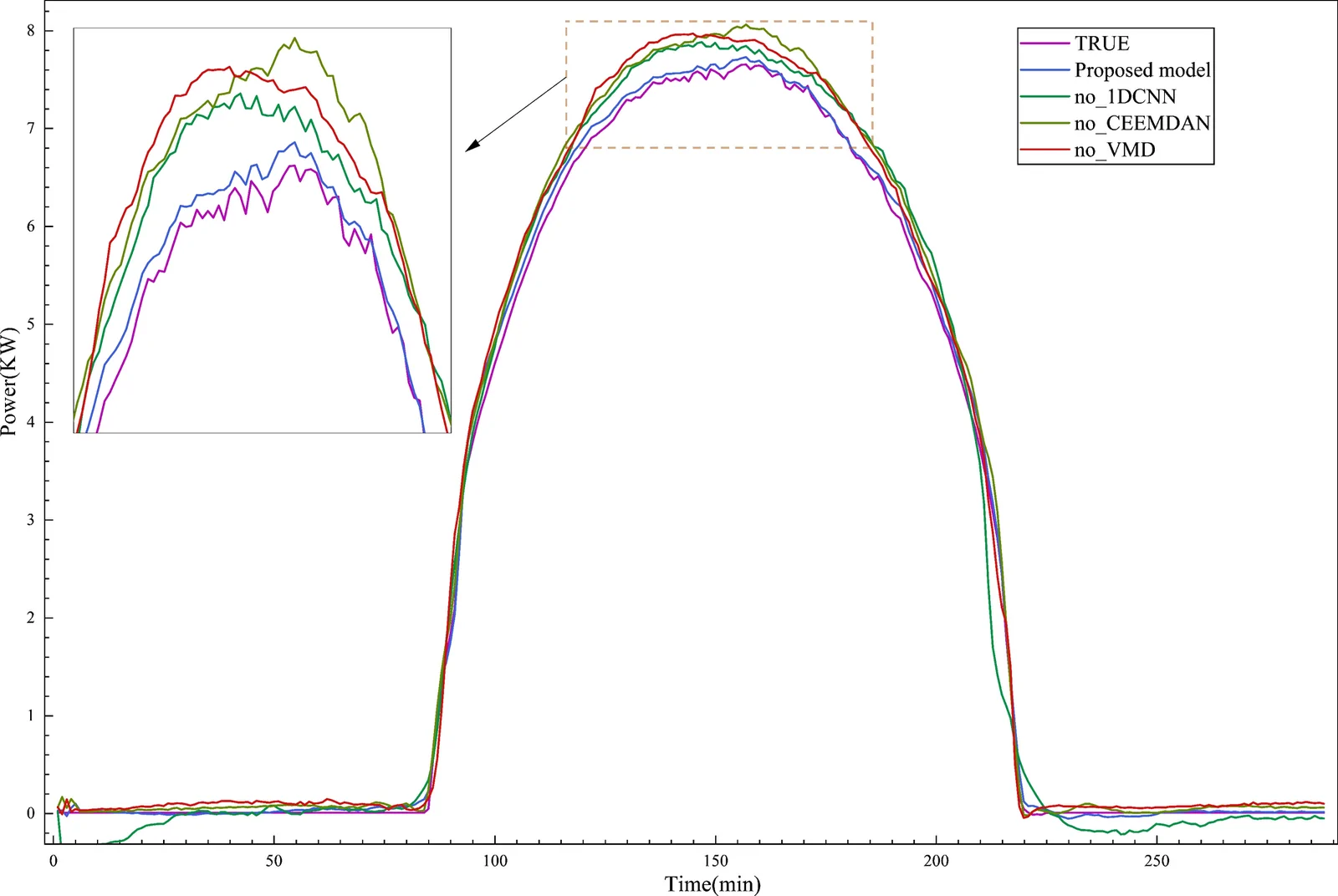
Predicted PV power curves of ablation variants vs. ground truth under the Sunny conditions.

**Fig. 10:**
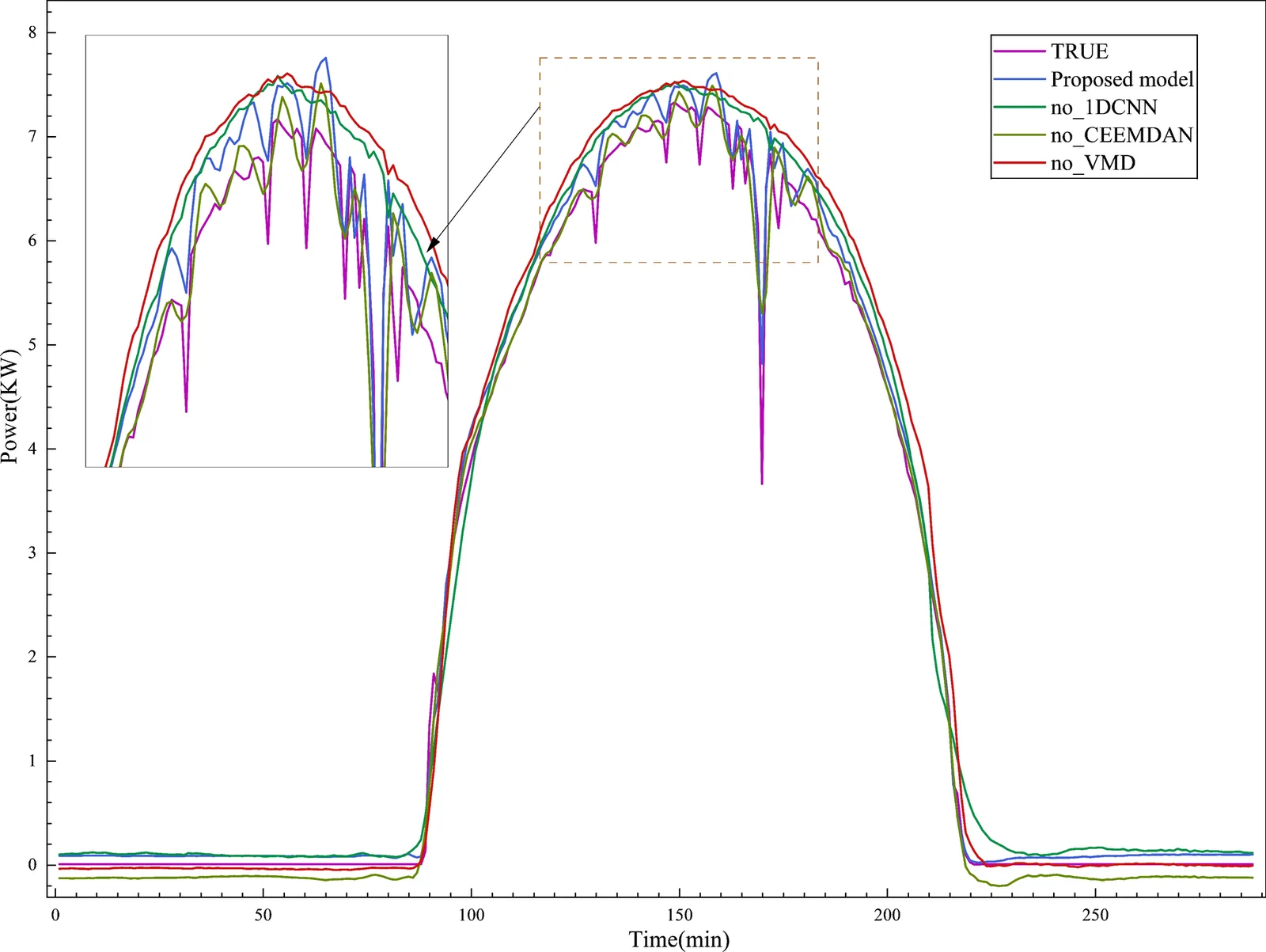
Predicted PV power curves of ablation variants vs. ground truth under the Cloudy conditions.

**Fig. 11:**
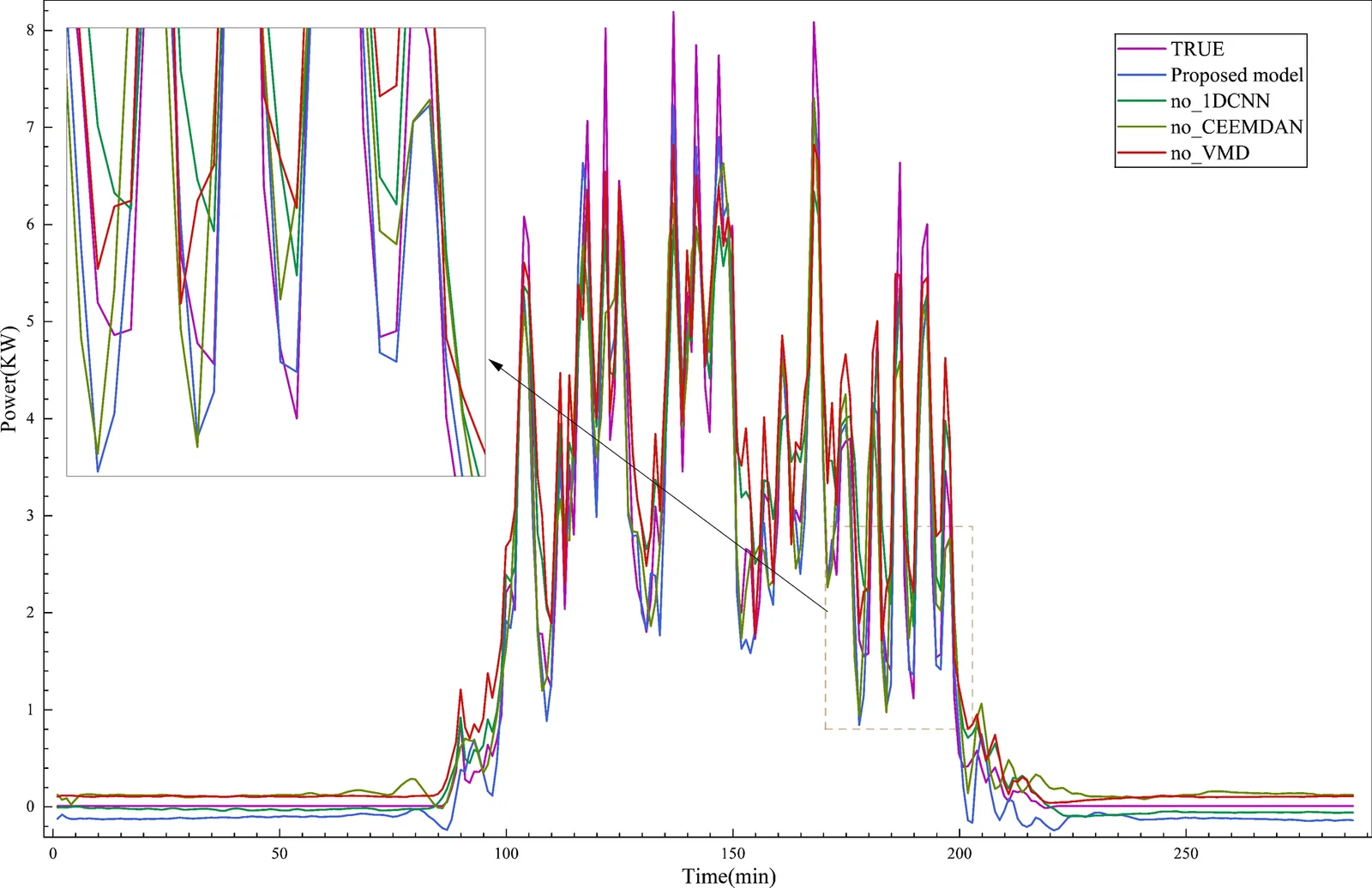
Predicted PV power curves of ablation variants vs. ground truth under the Rainy conditions.

To intuitively illustrate the performance differences, the predicted PV power curves of various ablation variants are compared against the ground truth in Figs. 9, 10 and 11 which are scenarios of a sunny-day case, a cloudy case, and a rainy case respectively.

- These predictions are outlined in Fig. 9 in the event of relatively quiet conditions (i.e., sunny or overcast conditions). The overall proposed model (VMD+CEEMDAN-1DCNN-S-Mamba) is perfectly matched to the ground truth curve and has extremely high peak value correlations and general trends. On the other hand, the model without VMD (CEEMDAN only) is significantly different especially in relation to the daily routine. The version without 1D-CNN module shows high smoothing properties and cannot maintain sharp edges indicating its inability to capture sufficient local variations.
- Figure 10 depicts a more sophisticated form of medium-level variations (cloudy weather). The given period is marked by a red box as it is a critical period, in which irradiance varies acutely. The proposed model overall remains highly faithful even in the situations of such drastic change. Nevertheless, the peak is predicted much smaller by the version of VMD-CEEMDAN-S-Mamba (no 1D-CNN) and it exhibits a delayed response, indicating that the 1D convolutional layers play a significant role in capturing short-term multiscale tendencies. This was expected, as the CEEMDAN-only model has severe delay and over-smoothing, which supports the notion of two-stage decomposition.
- Figure 11 demonstrates especially unstable conditions (rain or cloudy) when PV power is unpredictable and very variable. The performance gap is most pronounced in this highly volatile scenario. Despite its ability to follow the ground truth with good accuracy and remove noise effectively as well as retain significant oscillation. Simultaneously, the CEEMDAN alone and no-1D-CNN models are also extensively corrupted, too smooth, and fail to reflect the rapid change in power. With this visual output, it is very simple to realize that the removal of the two-stage decomposition or 1D-CNN module results in a considerable loss of tracking capability under complex weather conditions.

#### Analysis of ablation experiments

**Table 3:** Ablation experiment results of different models.

Decomposition strategy	Model architecture	MAE (kW)	RMSE (kW)	$R^2$
No VMD	CEEMDAN-1DCNN-S-Mamba	0.3053	0.5911	0.9424
No CEEMDAN	VMD-1DCNN-S-Mamba	0.1358	0.2749	0.9803
VMD+CEEMDAN	Proposed model	0.1188	0.2451	0.9822
No VMD+CEEMDAN	S-Mamba	0.3022	0.6035	0.9419

Table 3 presents the outcomes of the ablation experiments. The proposed optimal model (VMD-CEEMDAN-1DCNN-S-Mamba) demonstrates the best performance, achieving the lowest MAE (0.1188 kW) and RMSE (0.2451 kW), alongside the highest $R^2$ (0.9822). The results clearly indicate that different architectural strategies significantly impact the forecasting performance:

- Limitations of Single Decomposition: Relying solely on CEEMDAN yields subpar results (MAE = 0.3053 kW, $R^2$ = 0.9424), as it struggles to extract multi-scale characteristics from highly non-stationary series. Conversely, using only VMD (VMD-1DCNN-S-Mamba) significantly improves accuracy ($R^2$ reaches 0.9803) due to its superior coarse-grained frequency division. However, VMD alone fails to fully eliminate high-frequency residual noise, which disrupts subsequent feature extraction and limits further accuracy improvements.
- Superiority of Hybrid Decomposition: The proposed two-stage strategy resolves these limitations by utilizing VMD for preliminary coarse decomposition and CEEMDAN for fine-grained processing of residual and high-frequency components. Compared to the VMD-only model, this hybrid approach further reduces MAE by 12.5% and RMSE by 10.8%. This confirms that the synergy between VMD and CEEMDAN effectively mitigates modal aliasing and provides cleaner, adaptive multi-scale inputs.
- Indispensability of 1DCNN: When the 1DCNN module is removed (i.e., VMD-CEEMDAN-S-Mamba), the performance drastically degrades, with MAE surging to 0.3022 kW and $R^2$ dropping to 0.9419. This proves that 1DCNN is a critical bridging module. Directly feeding multi-channel decomposed signals into the S-Mamba without prior local feature extraction leads to severe feature redundancy and noise interference, negating the benefits of the preceding decomposition step.

In summary, the ablation study scientifically validates the necessity of the proposed structural design. No single algorithm can achieve optimal results independently. The integration of VMD-CEEMDAN multi-stage adaptive decomposition and 1DCNN deep feature fusion is imperative; they work synergistically to maximize the sequence modeling potential of the S-Mamba architecture, ultimately yielding the state-of-the-art predictive performance.

### Cross-validation

#### Robustness verification via 5-fold cross-validation

**Fig. 12:**
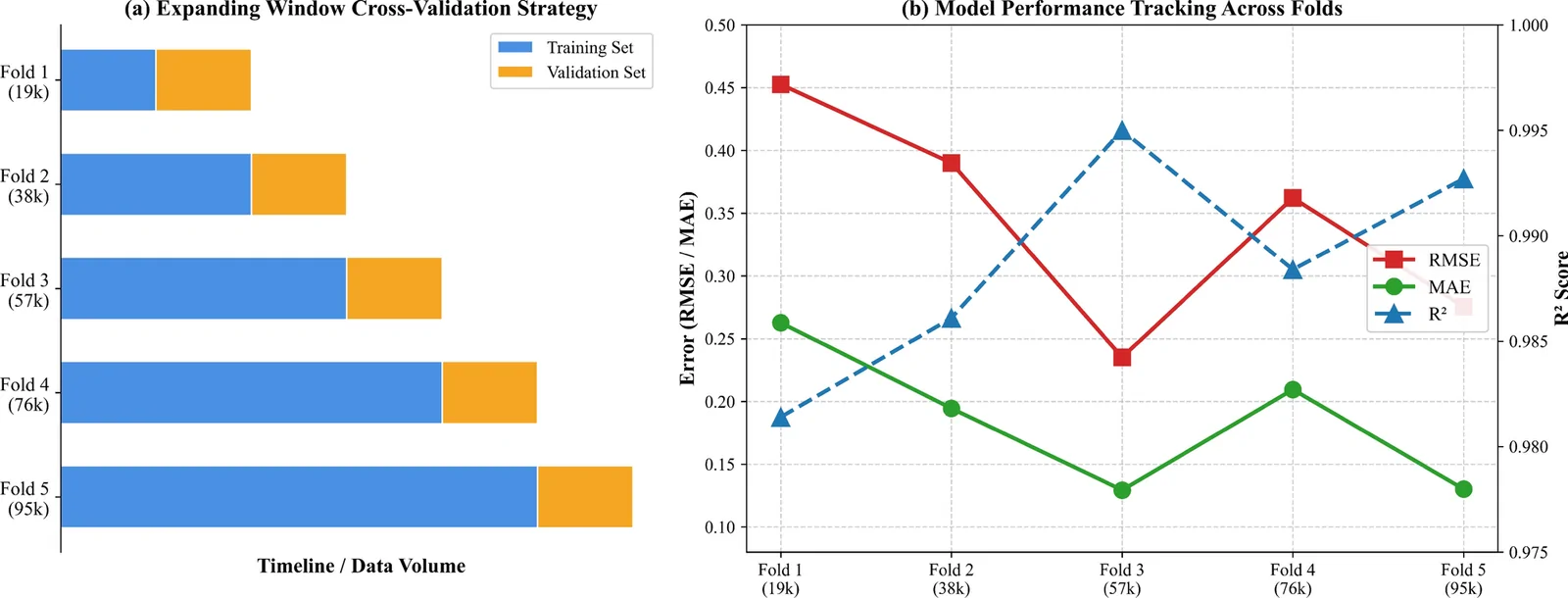
Robustness analysis via expanding window cross-validation.

To rigorously evaluate the generalization capability and temporal robustness of the proposed model, an expanding window cross-validation strategy was employed. Specifically, to establish a clear baseline of the model’s learning trajectory without the confounding variance of erratic weather transitions, this analysis was conducted exclusively on the “Sunny” dataset. Unlike standard k-fold cross-validation, which involves random data shuffling and consequently breaks the chronological continuity of time-series data, the expanding window approach mimics real-world deployment scenarios. It sequentially increments the training data volume while continuously shifting the validation window forward. The specific partition strategy and the corresponding evaluation results are illustrated in Fig. 12.

As depicted in Fig. 12a, the dataset was chronologically partitioned into five consecutive folds, with the training volume progressively expanding from 19,000 samples in Fold 1 to 95,000 samples in Fold 5. Figure 12b tracks the predictive metrics (RMSE, MAE, and $R^2$) across these folds. The overarching trajectory reveals a distinct positive correlation between training data availability and predictive performance. Specifically, from Fold 1 to Fold 3, the model exhibits a substantial decline in prediction errors, with the RMSE dropping from 0.4526 to 0.2352, and the $R^2$ score climbing from 0.9814 to a peak of 0.9950. This consistent improvement demonstrates the model’s excellent capacity to absorb larger volumes of historical data and effectively capture underlying long-term dependencies without suffering from overfitting.

However, a localized performance fluctuation is observed at Fold 4, where the RMSE temporarily rebounds to 0.3621. It is critical to note that in time-series expanding window validation, each fold’s validation set represents a strictly distinct chronological timeframe. A deeper inspection of the original dataset reveals that the specific validation period assigned to Fold 4 inherently contains a higher density of extreme peak values and anomalous fluctuations caused by real-world non-stationary factors. Despite facing this highly challenging and volatile validation subset, the proposed model still maintained a highly competitive $R^2$ score of 0.9884, indicating that its core feature-extraction mechanism remained stable and did not collapse under severe data shifts.

Furthermore, as the training window expanded to its maximum capacity in Fold 5, the model’s performance swiftly recovered, achieving an MAE of 0.1302 and an $R^2$ of 0.9927. To quantify the overall stability throughout the entire cross-validation process, the average metrics across all five folds were calculated. The model yielded an overall MAE of *0.1853 ± 0.0567* and an RMSE of *0.3431 ± 0.0877*. Most notably, the mean $R^2$ score reached an exceptional *0.9887 ± 0.0054*. The remarkably low variance in the $R^2$ score (*± 0.0054*) serves as strong quantitative evidence that the model’s predictive reliability remains highly consistent, regardless of the shifting validation windows. Ultimately, this experiment confirms that our model not only benefits from the scaling of training data but also maintains strong robustness against complex temporal anomalies.

#### Validation of spatial generalizability across multiple PV stations

A common limitation in data-driven forecasting research is the reliance on a single geographical location, which can restrict the universal applicability of the findings. To rigorously address concerns regarding spatial generalizability–and to verify that the proposed model does not merely overfit to the local microclimate of a single dataset–we expanded our evaluation to include three distinct and geographically independent PV power stations (denoted as Station A, Station B, and Station C). To ensure a comprehensive stress test, this multi-site validation was conducted across three diverse meteorological conditions: Sunny, Cloudy, and Rainy.

**Fig. 13:**
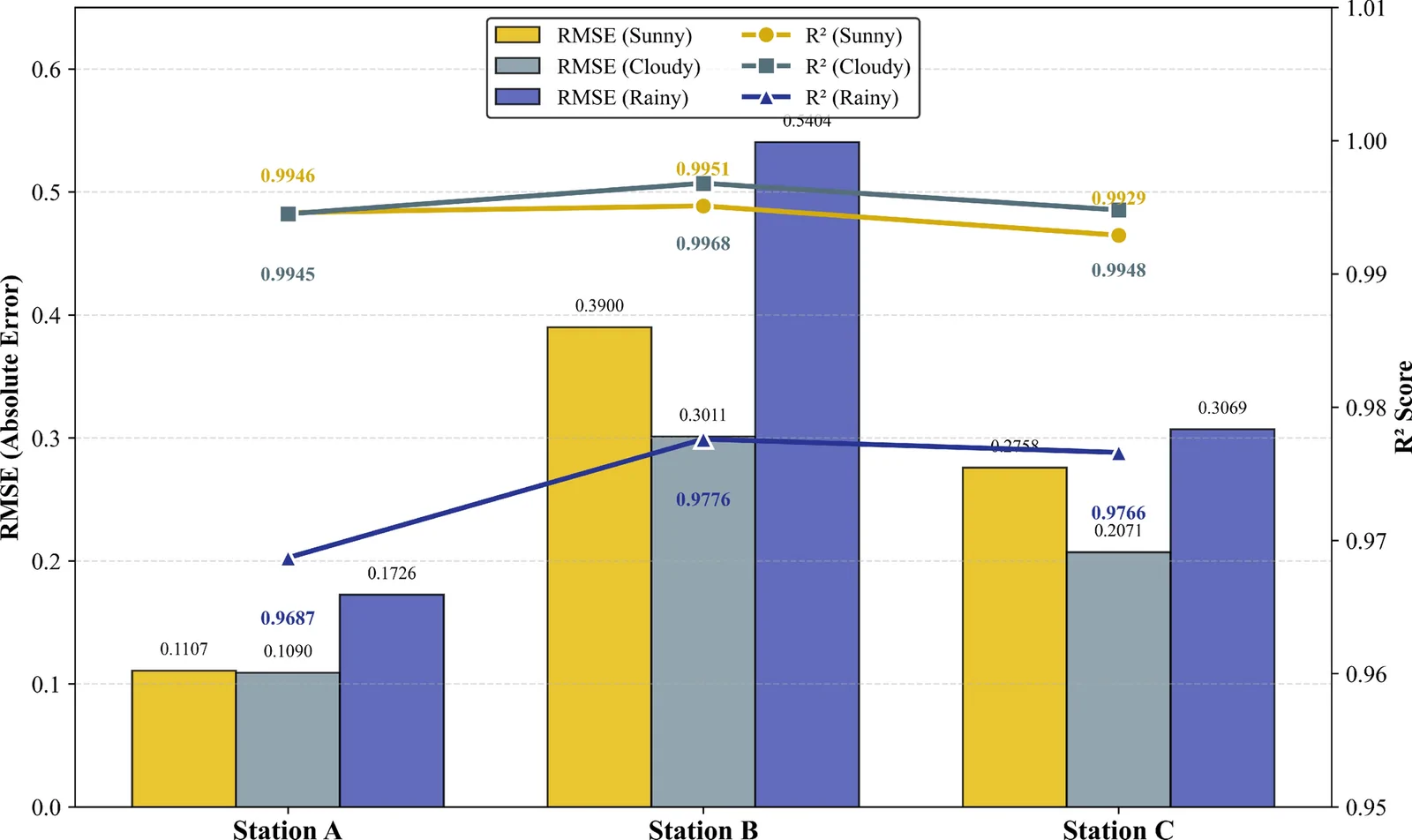
Comparison of prediction performance across three datasets.

**Table 4:** Prediction metrics for the three datasets under different weather conditions.

Station	Weather	MAE	RMSE	$R^2$
Station A	Sunny	0.0475	0.1107	0.9946
	Cloudy	0.0607	0.1090	0.9945
	Rainy	0.1272	0.1726	0.9687
Station B	Sunny	0.1757	0.3900	0.9951
	Cloudy	0.1453	0.3011	0.9968
	Rainy	0.3200	0.5404	0.9776
Station C	Sunny	0.1278	0.2758	0.9929
	Cloudy	0.1107	0.2071	0.9948
	Rainy	0.1770	0.3069	0.9766

The comparative results, as detailed in Fig. 13 and Table 4, provide strong evidence of the model’s universal applicability. The most prominent observation is the remarkable consistency of the evaluation metrics across all three independent stations. Under stable weather conditions (Sunny and Cloudy), the model consistently achieved $R^2$ scores exceeding 0.99 across the board. The slight variations in absolute errors (e.g., MAE and RMSE) among the stations are well within expected physical bounds, primarily driven by differences in each station’s installed capacity and the naturally reduced magnitude of power generation under cloud cover, rather than algorithmic instability.

More importantly, the true test of cross-site generalizability lies in the model’s response to highly volatile and stochastic environments. Under Rainy conditions, where intermittent cloud blockages severely disrupt standard generation curves, the absolute prediction errors predictably increased at all three locations (e.g., RMSE ranging from 0.1726 to 0.5404 depending on the station’s baseline). However, the critical indicator of generalizability is that the proposed algorithm maintained highly robust $R^2$ scores of 0.9687, 0.9776, and 0.9766 at Station A, B, and C, respectively. The fact that the performance did not suffer catastrophic degradation at any newly introduced site confirms that the model successfully learns the fundamental, universally applicable mapping between meteorological inputs and PV outputs, rather than memorizing site-specific noise.

In conclusion, this multi-station ablation effectively overcomes the limitations of single-site validation. The highly consistent performance baselines achieved across different geographical locations and diverse weather scenarios firmly establish that the proposed method possesses strong spatial generalizability, making it broadly applicable for PV forecasting tasks across various real-world environments.

### Robustness and statistical significance analysis

In deep learning-based time series forecasting, point estimates derived from a single training run may not sufficiently reflect the true generalization capability of a model due to the stochastic nature of weight initialization and the optimization process. To comprehensively evaluate the robustness of the proposed framework and eliminate potential bias introduced by randomness, the model was independently trained and tested 10 times under identical hyperparameter configurations. A rigorous statistical analysis was subsequently conducted to calculate the Mean, Standard Deviation (Std), 95% Confidence Interval (CI), and evaluate the statistical significance of the results.

The statistical outcomes regarding the mean and standard deviation across the 10 independent runs are summarized in Table 5. The exceptionally low standard deviations across all metrics explicitly demonstrate the remarkable stability and reproducibility of the proposed model. For instance, regarding the coefficient of determination ($R^2$), the standard deviations for the Sunny and Cloudy scenarios are merely 0.0008 and 0.0009, respectively. This indicates that the predictive accuracy of our framework is highly consistent and virtually impervious to random initialization.

**Table 5:** Statistical results of the proposed model across 10 independent runs (Mean ± Std).

Station	Weather	MAE	RMSE	$R^2$
Proposed Model	Sunny	0.0576 ± 0.0112	0.1232 ± 0.0159	0.9938 ± 0.0008
	Cloudy	0.0480 ± 0.0121	0.0855 ± 0.0288	0.9960 ± 0.0009
	Rainy	0.0942 ± 0.0531	0.1652 ± 0.0238	0.9709 ± 0.0093

To comprehensively address the statistical significance and confidence bounds of the proposed model’s performance, a detailed 95% Confidence Interval (CI) analysis was performed, as presented in Table 6.

**Table 6:** Detailed 95% Confidence Intervals (CI) for the evaluation metrics under distinct weather conditions.

Condition	Metric	Mean ± Std	95% CI
Sunny	MAE	0.0576 ± 0.0112	[0.0496, 0.0656]
	RMSE	0.1232 ± 0.0159	[0.1119, 0.1346]
	$R^2$	0.9938 ± 0.0008	[0.9932, 0.9943]
Cloudy	MAE	0.0480 ± 0.0121	[0.0393, 0.0566]
	RMSE	0.0855 ± 0.0288	[0.0650, 0.1061]
	$R^2$	0.9960 ± 0.0009	[0.9953, 0.9966]
Rainy	MAE	0.0942 ± 0.0531	[0.0562, 0.1322]
	RMSE	0.1652 ± 0.0238	[0.1481, 0.1822]
	$R^2$	0.9709 ± 0.0093	[0.9642, 0.9776]

The 95% Confidence Interval analysis provided in Table 6 yields compelling evidence of statistical significance. The extremely narrow confidence bounds, particularly for $R^2$ in the Sunny [0.9932, 0.9943] and Cloudy [0.9953, 0.9966] conditions, rigorously confirm that the state-of-the-art performance reported in the previous sections is statistically significant and reliable. This evaluation ensures that the superiority of our framework is intrinsically robust rather than a fortuitous occurrence.

## Discussion

This paper proposes a multi-step prediction approach to forecast photovoltaic (PV) power in the short term based on a combination of weather clustering, two-level signal decomposition, and hybrid deep learning. Initially, the framework clusters similar days using DTW + K-Medoids, then extracts multiscale features using VMD + CEEMDAN two-level decomposition, and finally feeds these results into a 1DCNN-S-Mamba hybrid model for prediction. It has been validated on a real-life dataset. The key findings and contributions include:

Historical data has been clustered using the DTW + K-Medoids algorithm into three typical weather types–sunny, cloudy, and rainy–and different predictive models have been developed accordingly. To confirm the effectiveness of this separation, t-SNE dimensionality reduction and visualization were applied. Implementing weather-specific modeling proved to be an extremely effective method for reducing the interference generated by diverse meteorological circumstances, which in turn made the model highly flexible and robust during complicated weather situations.

The hybrid decomposition scheme utilized a two-step process: VMD and CEEMDAN. Since this two-stage decomposition involves additional analysis of high-frequency signals, it is significantly more efficient in separating the various frequency components of the power sequences. This is particularly crucial for reducing the negative effects of the non-stationarity and non-linearity inherent in PV power signals. Experimental findings demonstrate that two-stage decomposition models yield considerably higher predictive accuracy than single-stage models based solely on VMD or CEEMDAN, and this benefit is especially evident under rainy conditions characterized by significant fluctuations.

Furthermore, the novel 1DCNN-S-Mamba hybrid forecasting model has been proposed. This architecture seamlessly integrates the local multi-scale feature extraction capability of 1DCNN with the S-Mamba model’s proficiency in capturing effective long-term time-series dependencies. By combining the Intrinsic Mode Functions (IMFs) and trend terms produced by the decomposition process with the original meteorological features, the model achieves a harmonious balance between advanced data preprocessing and deep learning. This synergy results in predictions that are substantially more accurate and consistent in multi-step scenarios.

The proposed model demonstrated excellent forecasting results across all three common weather conditions. The overall test set achieved an MAE of 0.1188 kW, an RMSE of 0.2451 kW, and an $R^2$ of 0.9822. Notably, the $R^2$ exceeded 0.99 during clear-sky (sunny) situations. Compared with eleven benchmark models, the proposed hybrid framework exhibited a significant improvement in MAE, RMSE, and $R^2$ values, fully validating the synergistic effectiveness of both the dual-layer decomposition and the hybrid network architecture.

Despite the superior forecasting performance of the proposed framework, several limitations must be acknowledged. First, the two-stage signal decomposition (VMD-CEEMDAN) coupled with the DTW distance calculation introduces a relatively high computational burden. While this complex preprocessing significantly enhances accuracy, the increased inference time may pose challenges for ultra-short-term, real-time online forecasting, especially when deployed on edge-computing devices with limited computational resources. Second, although the clustering strategy effectively handles sunny, cloudy, and rainy conditions, it may oversimplify transitional or extreme meteorological events (e.g., sudden snowstorms, heavy dust, or rapid passing clouds). The “hard clustering” approach might struggle to capture the nuances of days characterized by abrupt, intra-day weather shifts. Third, to ensure full transparency, it must be noted that a comprehensive sensitivity analysis for key hyperparameters–specifically the DTW window size, the VMD penalty factor *α*, and the CEEMDAN ensemble trials–was not performed in this study. Finally, like most data-driven deep learning approaches, the 1DCNN-S-Mamba model functions largely as a “black box,” lacking the physical interpretability found in traditional physical PV models. The framework does not explicitly account for physical degradation factors such as PV panel aging, surface soiling, or inverter efficiency loss over long periods.

Future work will focus on addressing these limitations. Immediate efforts will include conducting rigorous sensitivity analyses on the aforementioned key hyperparameters to further optimize the framework’s configuration and transparency. Additionally, efforts will be made to optimize the decomposition algorithms to reduce computational complexity without sacrificing accuracy, potentially exploring lightweight end-to-end architectures. Furthermore, incorporating spatial-temporal correlations from neighboring PV stations using Graph Neural Networks (GNNs) and integrating physical equations (Physics-Informed Neural Networks) could further enhance the model’s interpretability and robustness under extreme weather conditions.

## Methods

### Data description and preprocessing

#### Data sources

In this study, the operational data used to validate the proposed forecasting framework was collected from the Desert Knowledge Australia Solar Centre (DKASC), a highly recognized public PV testing facility located in Alice Springs, Australia. To comprehensively evaluate the generalization capability and robustness of the model across different hardware configurations, three distinct PV installations–designated as Station A, Station B, and Station C–were selected as the research subjects. The dataset spans a continuous two-year period from September 1, 2013, to September 1, 2015.

**Table 7:** Technical specifications of the selected PV stations from the DKASC dataset.

Station	Manufacturer	Capacity (kW)	Cell technology	Tracking type	Year
Station A	Trina	10.5	Mono-Si	Dual-axis	2009
Station B	eco-Kinetics	26.5	Mono-Si	Dual-axis	2010
Station C	BP Solar	5.0	Poly-Si	Fixed (Roof Mounted)	2008

As detailed in Table 7, these three stations were deliberately chosen to encompass a diverse range of technical specifications. They vary significantly in rated capacity (ranging from 5.0 kW to 26.5 kW), PV module technology (monocrystalline silicon vs. polycrystalline silicon), and tracking systems (dual-axis tracking vs. fixed-tilt/roof-mounted arrays). By utilizing data from such heterogeneous physical environments, the study effectively demonstrates that the proposed clustering-decomposition-deep learning framework is not biased toward a specific PV technology or installation method, thereby verifying its broad applicability and consistency in real-world scenarios. The temporal resolution of the collected power and meteorological data is 5 minutes (Table 8).

#### Features

**Table 8:** Description of the variables in the utilized PV dataset.

Category	Feature name	Description	Unit
Temporal	timestamp	Date and time of the observation (5-min interval)	–
Electrical	Active_Power	Instantaneous active power generation	kW
	Active_Energy_Delivered_Received	Cumulative energy delivered to the grid	kWh
	Current_Phase_Average	Average current across phases	A
	Performance_Ratio	Ratio of actual to theoretical energy output	%
Meteorological	Weather_Temperature_Celsius	Ambient weather temperature	$^\circ$C
	Weather_Relative_Humidity	Relative humidity of the air	%
	Wind_Speed	Speed of the wind	m/s
	Wind_Direction	Direction of the wind (0-360 degrees)	$^\circ$
	Weather_Daily_Rainfall	Accumulated rainfall for the day	mm
Irradiance	Global_Horizontal_Radiation	Global Horizontal Irradiance (GHI)	W/m$^2$
	Diffuse_Horizontal_Radiation	Diffuse Horizontal Irradiance (DHI)	W/m$^2$
	Radiation_Global_Tilted	Global Tilted Irradiance (Plane of Array)	W/m$^2$
	Radiation_Diffuse_Tilted	Diffuse Tilted Irradiance on the array plane	W/m$^2$

#### Data preprocessing

In real-world photovoltaic (PV) systems, the collected raw data often contains missing values due to sensor failures or communication network interruptions. Furthermore, the features in the dataset exhibit significantly different scales and units (e.g., solar radiation in W/m$^2$ and temperature in $^\circ$C). To ensure the continuity of the time series and improve the convergence speed of the predictive model, a two-step data preprocessing strategy is applied.

Since the PV dataset is a continuous time series recorded at 5-minute intervals, missing values are predominantly short-term gaps. Therefore, linear interpolation is employed to estimate the missing data points. This method constructs a straight line between the nearest valid data points before and after the missing timestamp. The mathematical expression is defined as follows:

4 $$\begin{aligned} y = y_0+\frac{y_1 - y_0}{t_1 - t_0} (t - t_0) \end{aligned}$$

where: *t* represents the timestamp of the missing data point. *y* denotes the interpolated feature value at time *t*. $t_0$ and $t_1$ are the timestamps of the nearest valid observations immediately preceding and succeeding the missing point, respectively. $y_0$ and $y_1$ represent the actual recorded feature values at times $t_0$ and $t_1.$ The variance in magnitudes across different features can cause gradient descent-based algorithms to converge slowly or become biased toward features with larger numerical values. To eliminate the dimensional influence, Min-Max normalization is applied to linearly transform each feature into a bounded range, typically [0, 1]. The normalization formula is given by:

5 $$\begin{aligned} x_{norm} = \frac{x - x_{min}}{x_{max} - x_{min}} \end{aligned}$$

where: $x_{norm}$ is the normalized value of the feature. *x* is the original observed value. $x_{min}$ denotes the minimum value of the specific feature across the training dataset. $x_{max}$ represents the maximum value of the feature across the training dataset.It is worth noting that the statistical parameters (*μ* and *σ*) are computed exclusively from the training set to prevent data leakage during the validation and testing phases.

### Weather pattern clustering

#### Dynamic time warping (DTW) for similarity measurement

In real-world scenarios, the movement of clouds causes temporal shifts and delays in meteorological features and solar irradiance. For instance, a sudden drop in PV power due to cloud cover might occur at 10:00 AM on one day but at 10:30 AM on another. Traditional Euclidean distance performs strict one-to-one point alignment on the time axis, which fails to capture such temporal deformations.

To address this, Dynamic Time Warping (DTW) is employed to measure the similarity between time series. DTW dynamically aligns two sequences by allowing non-linear mapping along the time axis, effectively compensating for time-shifts. Given two daily time series sequences $X = (x_1, x_2,..., x_n)$ and $Y = (y_1, y_2,..., y_m)$, the core state-transition equation (dynamic programming approach) to calculate the accumulated cost matrix *D*(*i*, *j*) is defined as:

6 $$\begin{aligned} D(i,j) = d(x_i, y_j) + \min \left\{ \begin{matrix} D(i-1, j) \\ D(i, j-1) \\ D(i-1, j-1) \end{matrix} \right\} \end{aligned}$$

where: *D*(*i*, *j*) represents the minimal accumulated distance from the starting point (1, 1) to the current alignment point (*i*, *j*). $d(x_i, y_j)$ is the local distance (typically Euclidean distance) between the data point $x_i$ in sequence *X* and $y_j$ in sequence *Y*. $\min \{\cdot \}$ denotes the optimal previous path selection, representing insertion, deletion, and match operations, respectively.

#### Robust clustering using K-Medoids

While K-Means is a widely used clustering algorithm, it updates its cluster centers by calculating the mean of the samples. This makes it highly sensitive to extreme outliers, such as a sudden, massive dark cloud temporarily dropping the PV output to near zero. These anomalies can drag the calculated mean away from the main distribution, creating “virtual” cluster centers that do not exist in the actual physical world.

To improve robustness, the K-Medoids algorithm is utilized. Instead of computing an artificial mean, K-Medoids strictly selects an actual, existing data point from the dataset as the cluster center (medoid). By coupling K-Medoids with the DTW distance metric, the objective function aims to minimize the sum of DTW distances between each daily sequence and its corresponding medoid:

7 $$\begin{aligned} J = \sum _{k=1}^{K} \sum _{X_i \in C_k} \text {DTW}(X_i, M_k) \end{aligned}$$

where: *J* is the total cost function to be minimized. *K* is the predefined number of clusters (in this study, *K=3*). $C_k$ represents the set of all time series assigned to the *k*-th cluster. $X_i$ is a daily time series sample belonging to cluster $C_k$. $M_k$ is the selected medoid (an actual representative sequence) for the *k*-th cluster. $\text {DTW}(X_i, M_k)$ calculates the dynamic time warping distance between sample $X_i$ and medoid $M_k.$

#### Clustering results and validation

To validate the effectiveness of the proposed DTW + K-Medoids clustering strategy, the results are analyzed from both feature space and physical interpretation perspectives.

**Fig. 14:**
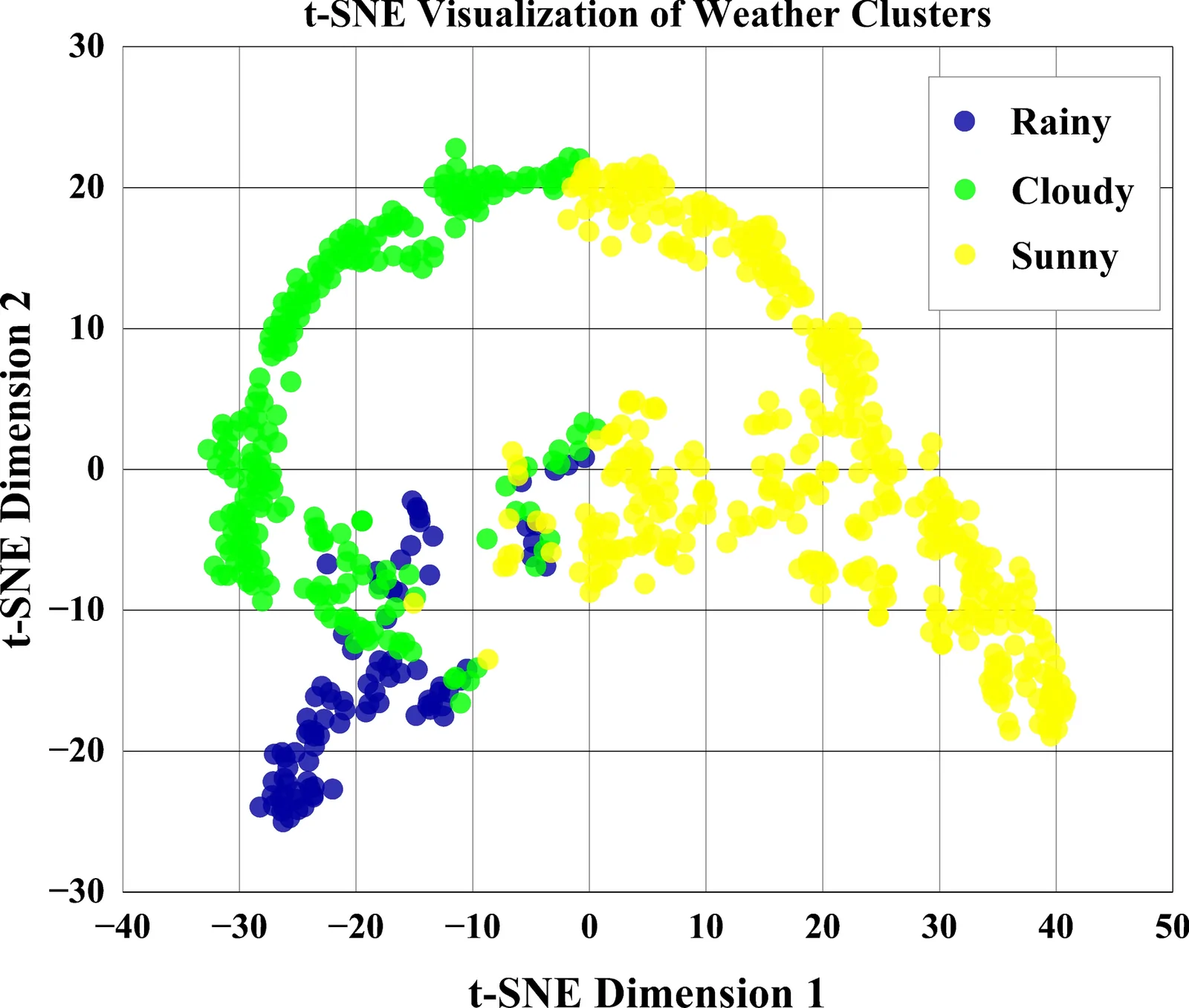
t-SNE visualization of the weather clustering results.

First, t-Distributed Stochastic Neighbor Embedding (t-SNE) is applied to map the high-dimensional time-series features into a 2D space. As depicted in Fig. 14, the samples are distinctly grouped into three well-separated clusters, demonstrating that the algorithm successfully differentiates the underlying meteorological patterns regardless of temporal shifts and outliers.

**Fig. 15:**
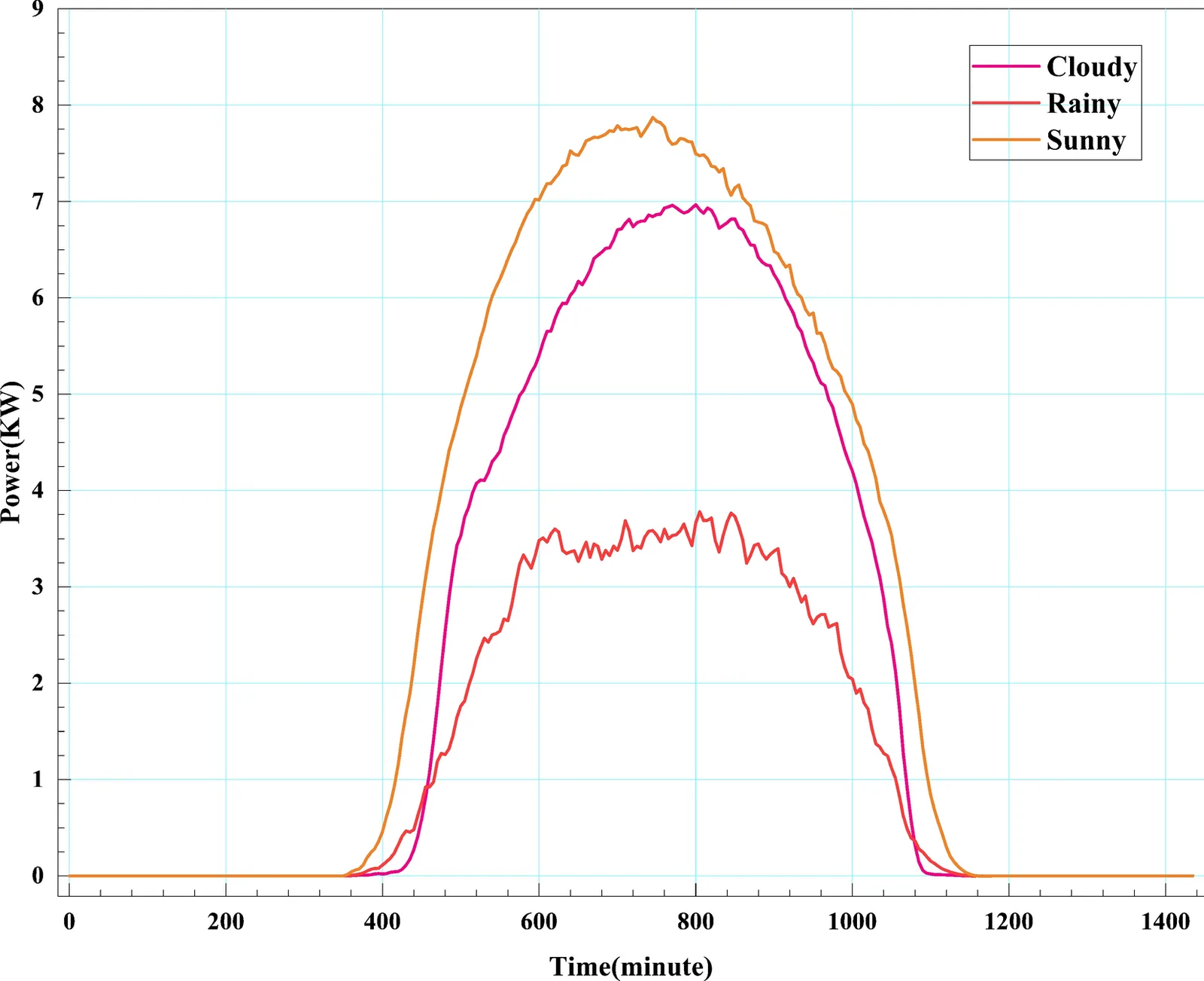
Mean power curves for the three weather clusters.

Second, to verify the physical significance of the established clusters, the average daily PV power generation curves for the three identified categories are plotted in Fig. 15. The resultant profiles highly align with real-world weather characteristics: the ’Sunny’ cluster exhibits a smooth curve with the highest peak generation; the ’Cloudy’ cluster shows moderate generation capacity; and the ’Rainy’ cluster displays the lowest power output accompanied by severe high-frequency fluctuations. These distinct power curve shapes confirm the rationality and accuracy of the clustering approach.

### Two-stage signal decomposition: VMD and CEEMDAN

#### First-layer decomposition: VMD for dominant trends

Variational Mode Decomposition (VMD) is a non-recursive, adaptive signal processing method. Unlike the traditional Empirical Mode Decomposition (EMD) which is prone to mode aliasing (mode mixing), VMD explicitly formulates the decomposition as a constrained variational problem. It concurrently extracts *K* discrete band-limited modes (IMFs) while minimizing the sum of their estimated bandwidths.

For a given original photovoltaic power series *f*(*t*), the core constrained variational problem is mathematically defined as:

8 $$\begin{aligned} \begin{aligned}&\min _{\{u_k\}, \{\omega _k\}} \left\{ \sum _{k=1}^K \left\| \partial _t \left[ \left( \delta (t) + \frac{j}{\pi t} \right) * u_k(t) \right] e^{-j\omega _k t} \right\| _2^2 \right\} \\&\text {s.t.} \quad \sum _{k=1}^K u_k(t) = f(t) \end{aligned} \end{aligned}$$

where: $u_k(t)$ represents the k-th intrinsic mode function (IMF). $\omega _k$ denotes the central frequency corresponding to the k-th mode. *δ (t)* represents the Dirac distribution. * denotes the convolution operator.To solve this constrained optimization problem, the quadratic penalty factor *α* and the Lagrangian multiplier *λ (t)* are introduced. The penalty factor guarantees reconstruction fidelity in the presence of Gaussian noise, while the Lagrangian multiplier strictly enforces the constraints. The problem is thus transformed into an unconstrained augmented Lagrangian function $\mathcal {L}$, expressed as:

9 $$\begin{aligned} \begin{aligned} \mathcal {L}(\{u_k\}, \{\omega _k\}, \lambda ) =&\ \alpha \sum _{k=1}^K \left\| \partial _t \left[ \left( \delta (t) + \frac{j}{\pi t} \right) * u_k(t) \right] e^{-j\omega _k t} \right\| _2^2 \\&+ \left\| f(t) - \sum _{k=1}^K u_k(t) \right\| _2^2 + \left\langle \lambda (t), f(t) - \sum _{k=1}^K u_k(t) \right\rangle \end{aligned} \end{aligned}$$

The core of VMD optimization lies in employing the Alternating Direction Method of Multipliers (ADMM) to iteratively update the modes $u_k$, central frequencies $\omega _k$, and Lagrangian multiplier *λ*. By transforming the signals into the frequency domain via Fourier transform, the specific update expressions for the *(n+1)*-th iteration are as follows:

10 $$\begin{aligned} \hat{u}_k^{n+1}(\omega ) = \frac{\hat{f}(\omega ) - \sum _{i \ne k} \hat{u}_i(\omega ) + \frac{\hat{\lambda }(\omega )}{2}}{1 + 2\alpha (\omega - \omega _k)^2} \end{aligned}$$

11 $$\begin{aligned} \omega _k^{n+1} = \frac{\int _0^\infty \omega |\hat{u}_k^{n+1}(\omega )|^2 d\omega }{\int _0^\infty |\hat{u}_k^{n+1}(\omega )|^2 d\omega } \end{aligned}$$

12 $$\begin{aligned} \hat{\lambda }^{n+1}(\omega ) = \hat{\lambda }^n(\omega ) + \tau \left( \hat{f}(\omega ) - \sum _{k=1}^K \hat{u}_k^{n+1}(\omega ) \right) \end{aligned}$$

where: $\hat{u}(\omega )$, $\hat{f}(\omega )$ and $\hat{\lambda }(\omega )$ represent the Fourier transforms of their corresponding time-domain signals. *τ* denotes the update step size for the dual ascent.Through this strict mathematical framework, VMD completely avoids mode aliasing and demonstrates superior performance in extracting low-frequency, trend-dominant signals. Following the decomposition process, the lower-order modes (e.g., IMF_1 and IMF_2) can smoothly and accurately capture the macroscopic diurnal cycle of solar generation.

#### Second-layer decomposition: CEEMDAN for high-frequency residuals

While VMD exhibits superior performance in extracting low-frequency deterministic trends due to its band-limited assumption, it fundamentally struggles to process broad-band, highly non-stationary stochastic signals. Consequently, after the first-layer VMD, a high-frequency residual component (labeled as ‘residual‘ in Fig. 2) is inevitably generated. This residual is not mere random noise; it encapsulates crucial transient physical phenomena, such as sudden irradiance drops caused by rapid cloud movement. Discarding or feeding this highly volatile residual directly into predictive models would severely degrade forecasting accuracy, particularly under severe weather conditions.

To resolve this, Complete Ensemble Empirical Mode Decomposition with Adaptive Noise (CEEMDAN) is employed to perform a “secondary fine-grained dissection” exclusively on this high-frequency residual. CEEMDAN effectively addresses the mode-mixing problem of traditional EMD and the incomplete reconstruction issue of EEMD by adding finite adaptive white noise at each stage and calculating a unique residue.

Let the high-frequency residual obtained from VMD be denoted as $r_{vmd}(t)$. The CEEMDAN decomposition process is mathematically formalized as follows: First, a specific realization of white noise $w^i(t)$ with a controlled amplitude $\varepsilon _0$ is added to the target signal. The first IMF is obtained by averaging the first modes extracted by standard EMD over *N* ensemble trials:

13 $$\begin{aligned} IMF_1(t) = \frac{1}{N} \sum _{i=1}^{N} E_1 \left( r_{vmd}(t) + \varepsilon _0 w^i(t) \right) \end{aligned}$$

where $E_k(\cdot )$ operates as a function to extract the *k*-th mode using standard EMD. The first sequential residue is then calculated as $R_1(t) = r_{vmd}(t) - IMF_1(t)$.

For the subsequent *k*-th mode (*k ≥ 2*), adaptive noise is added to the previous residue $R_{k-1}(t)$. The *k*-th IMF is calculated as:

14 $$\begin{aligned} IMF_k(t) = \frac{1}{N} \sum _{i=1}^{N} E_1 \left( R_{k-1}(t) + \varepsilon _{k-1} E_{k-1}(w^i(t)) \right) \end{aligned}$$

The algorithm iteratively computes $R_k(t) = R_{k-1}(t) - IMF_k(t)$ until the obtained residue contains less than two extrema. The final complete reconstruction is rigorously defined as:

15 $$\begin{aligned} r_{vmd}(t) = \sum _{k=1}^{K} IMF_k(t) + R_{final}(t) \end{aligned}$$

where $R_{final}(t)$ is the ultimate stationary trend of the residual.

The core rationale behind splicing VMD and CEEMDAN lies in exploiting their *complementary mathematical properties in the frequency domain*. Applying empirical decomposition (like EMD/CEEMDAN) directly to the raw PV signal often generates an excessive number of sub-modes and suffers from endpoint effects due to the strong diurnal periodicity. Conversely, relying solely on VMD fails to decipher the complex, non-stationary weather fluctuations hidden in the high-frequency spectrum.

The proposed dual-layer architecture explicitly resolves this paradox through a “divide and conquer” frequency-division strategy:

- *Macroscopic Trend Isolation:* VMD acts as a strict mathematical band-pass filter, peeling off the regular, large-amplitude diurnal cycles (low-frequency IMFs). This drastically reduces the complexity of the original sequence.
- *Microscopic Volatility Anatomy:* CEEMDAN is strategically applied *only* to the chaotic, broad-band VMD residual. **Through this fine-grained decomposition process**, the previously tangled high-frequency noise is successfully disentangled into several subtle, **yet** predictable sub-modes (e.g., IMF_1 to IMF_3).

By splicing these two algorithms, the framework ensures absolute feature purity–providing smooth inputs for identifying daily patterns while offering cleanly separated high-frequency features for capturing transient meteorological changes. This thorough decomposition is fundamentally vital for enhancing the deep learning network’s capacity to predict power generation under complex, highly variable weather types.

### 1DCNN-S-Mamba

After the dual-layer decomposition using VMD-CEEMDAN, the non-stationary PV sequence is transformed into a set of relatively stable intrinsic mode functions. However, to achieve highly accurate predictions, the deep learning model must possess the capability to simultaneously capture two distinct types of data dynamics: Local abrupt features (e.g., sudden irradiance drops caused by fast-moving clouds) and long-range temporal dependencies (e.g., the macroscopic diurnal cycle and multi-day weather inertia).

**Fig. 16:**
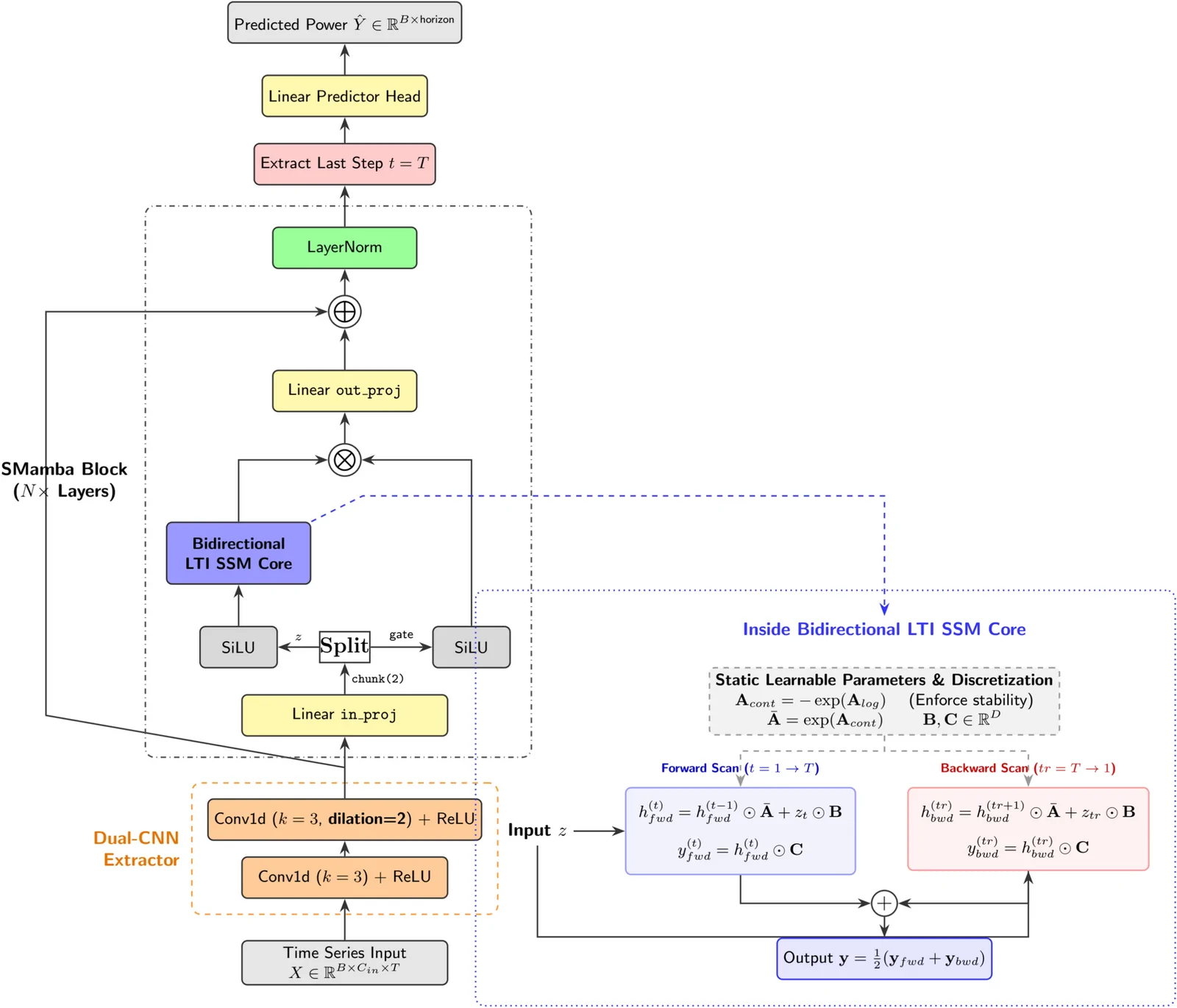
The detailed architecture of the proposed 1DCNN-S-Mamba model.

To satisfy both requirements without incurring massive computational overhead, we propose a hybrid feature extractor combining a 1D Convolutional Neural Network (1D-CNN) and an S-Mamba block. The overall architecture of the proposed model is illustrated in Fig. 16. The division of labor between these two components is highly complementary.

#### 1D-CNN for local volatility extraction

The first stage of the network utilizes a Dual-1D-CNN. In the context of time-series forecasting, 1D convolution operations act as highly efficient sliding windows across the temporal dimension. Their primary role is to focus on restricted receptive fields to capture adjacent, localized abrupt changes. To expand the receptive field without increasing parameters, we introduce a dilated convolution in the second layer.

Mathematically, given the input sequence $\textbf{X} \in \mathbb {R}^{B \times C_{in} \times T}$, the dilated 1D convolution operation is formulated as:

16 $$\begin{aligned} \textbf{Z}_c[t] = \text {ReLU} \left( \sum _{k=0}^{K-1} \textbf{W}_c[k] \cdot \textbf{X}[t - d \cdot k] + \textbf{b}_c \right) \end{aligned}$$

where: *K=3* denotes the kernel size. $\textbf{W}_c$ and $\textbf{b}_c$ are the learnable weights and biases. d represents the dilation rate (set to 2 in the second layer). $\textbf{Z}_c$ denotes the extracted local feature representation.

#### S-Mamba for long-range dependency modeling

Addressing the Bottlenecks of Traditional Models: While 1D-CNN is adept at local features, it lacks the global receptive field required to understand long-term historical context. Traditionally, Recurrent Neural Networks (like LSTMs) inherently suffer from gradient vanishing over long sequences. Transformers rely on self-attention mechanisms with a computational complexity of $\mathcal {O}(L^2)$ with respect to the sequence length *L*, creating an unacceptable memory bottleneck for high-resolution forecasting.

To overcome this, we construct the S-Mamba block. Rooted in structured State Space Models (SSMs), Mamba achieves an infinite-context global receptive field with strictly linear $\mathcal {O}(L)$ complexity. To better adapt to the relatively stable periodic physical laws of PV time series, our S-Mamba employs a static Linear Time-Invariant (LTI) core combined with a bidirectional scan mechanism, avoiding the over-fitting risks associated with highly dynamic data-dependent parameters.

To quantitatively demonstrate this structural advantage, Table 9 provides a direct empirical comparison between a standard Transformer block and our proposed S-Mamba block. Evaluated under the unified hardware environment detailed previously (48 GB vGPU), S-Mamba exhibits significant reductions in computational overhead, training time, and inference latency, effectively resolving the memory and computational bottlenecks associated with traditional attention mechanisms.

**Table 9:** Computational complexity and efficiency comparison. *L* denotes sequence length, *D* is the hidden dimension, and *N* represents the SSM state dimension.

Model architecture	Theoretical complexity	Training time / batch	Training time / epoch
Transformer-based	$\mathcal {O}(L^2 \cdot D)$	15.68 ms	160.71 s
S-Mamba	$\mathcal {O}(L \cdot D \cdot N)$	9.07 ms	129.52 s

As shown in Table 9, our S-Mamba block not only reduces the computational FLOPs but also requires significantly fewer parameters, proving it to be a highly lightweight and memory-efficient architecture for PV forecasting.

Mathematical Formulation: Inside the S-Mamba block, the input sequence is first split into two branches: a main feature branch *z* and a gating branch $\text {gate}$. For the main branch, we define global static learnable parameters $\textbf{A}_{log}, \textbf{B}, \textbf{C} \in \mathbb {R}^{D}$. To ensure the stability of the state transition, the continuous transition matrix is constrained as $\textbf{A}_{cont} = -\exp (\textbf{A}_{log})$, and then discretized:

17 $$\begin{aligned} \bar{\textbf{A}} = \exp (\textbf{A}_{cont}) \end{aligned}$$

To overcome the causal restriction (unidirectional view) of standard SSMs, S-Mamba performs a bidirectional sequence scan. For time step *t* in the forward scan and step *tr* in the backward scan, the hidden states *h* are updated via Hadamard product (*⊙*) as follows:

18 $$\begin{aligned} \begin{aligned} h_{fwd}^{(t)}&= h_{fwd}^{(t-1)} \odot \bar{\textbf{A}} + z_t \odot \textbf{B} \\ h_{bwd}^{(tr)}&= h_{bwd}^{(tr+1)} \odot \bar{\textbf{A}} + z_{tr} \odot \textbf{B} \end{aligned} \end{aligned}$$

The final output of the bidirectional LTI SSM core is obtained by averaging the projected responses from both directions:

19 $$\begin{aligned} \textbf{y}_{ssm} = \frac{1}{2} (h_{fwd} \odot \textbf{C} + h_{bwd} \odot \textbf{C}) \end{aligned}$$

Finally, to control the information flow, S-Mamba utilizes a multiplicative gating mechanism. The SSM output is modulated by the non-linear activation of the gating branch: $\textbf{y}_{out} = \textbf{y}_{ssm} \otimes \text {SiLU}(\text {gate})$. This architecture robustly captures the long-term meteorological context while maintaining high computational efficiency.

### Parameter configuration and implementation details

To ensure the reproducibility of the proposed framework and achieve optimal performance, all hyperparameters were carefully configured based on empirical testing and grid search strategies. The complete implementation is developed using Python 3 and the PyTorch deep learning framework. The parameter configurations are detailed below across four main modules: time-series clustering, dual-stage signal decomposition, sequence modeling architecture, and model training.

#### Time-series clustering (DTW-K-Medoids)

For the weather condition clustering module, the Dynamic Time Warping (DTW) algorithm was applied with a continuous constraint window size of *w = 30* and early pruning enabled to accelerate distance matrix computation. Based on the calculated DTW distance matrix, the K-Medoids clustering algorithm was deployed using the FasterPAM optimization method. The number of clusters was predetermined to *K = 3* (representing Sunny, Cloudy, and Rainy). The maximum number of iterations was set to 300, and the initialization method was set to build with a fixed random seed of 42 to guarantee deterministic assignments.

#### Dual-stage decomposition (VMD-CEEMDAN)

To extract both global patterns and local volatility from the PV power sequences, a dual-stage decomposition strategy was configured:

VMD Configuration: The secondary penalty factor (bandwidth parameter) *α* was set to 2000 to prevent mode mixing, while the noise tolerance *τ* was set to 0 (assuming strict data fidelity). The number of Intrinsic Mode Functions (IMFs) was designated as *K = 5*. The initial center frequencies were uniformly distributed (init = 1), with a convergence tolerance of $1 \times 10^{-7}$.

CEEMDAN Configuration: Applied strictly to the VMD residual sequences, the ensemble trials were set to 50 to balance computational efficiency and decomposition robustness. The white noise standard deviation ratio (epsilon) was configured at 0.05, with a noise scale of 0.2. The maximum number of IMFs was determined automatically.

#### 1DCNN-S-Mamba architecture

The proposed deep learning core handles feature extraction and sequence modeling. The look-back window size was fixed at 30 steps to predict the future 3 steps (Direct multi-step forecasting). The network configurations are as follows:

Feature Extraction (1D-CNN): The spatial feature extractor consists of two 1D convolutional layers. Both layers utilize a kernel size of 3 and a hidden dimension of 64. To expand the receptive field without losing resolution, the second convolutional layer employs a dilation rate of 2. The ReLU activation function is applied immediately after each convolutional operation.

Sequence Modeling (S-Mamba): The temporal dependencies are captured by 2 stacked Selective Mamba blocks. The hidden dimension is maintained at 64.

Internal Mechanisms: An expansion factor of *E=2* is applied, projecting the inner dimension to 128.

Activations and Projections: Inside the block, the inputs are split into two parallel paths (state transitions and gating mechanisms), activated by the SiLU (Swish) function before element-wise multiplication.

State Space Parameters: The state transition matrix parameters $\textbf{B}$ and $\textbf{C}$ are initialized from a normal distribution with a standard deviation of 0.02. The continuous log-scale parameter $\textbf{A}_{log}$ is initialized at $\ln (10^{-2})$.

Regularization and Normalization: To prevent gradient vanishing and ensure training stability, Residual Connections are added across each Mamba block, followed immediately by a Layer Normalization (LayerNorm) layer. No explicit Dropout layer is utilized, as LayerNorm and the gating mechanism inherently regularize the representation.

#### Model training settings

For model optimization, both features and targets were scaled using MinMaxScaler bounded between 0 and 1. The model was trained using the AdamW optimizer with an initial learning rate of 0.001. The MSELoss (Mean Squared Error) was utilized as the objective function, and the batch size was set to 32. To effectively prevent overfitting and ensure optimal generalization, an early stopping mechanism was integrated into the training process. The training epochs were dynamically determined within a range of 10 to 100 epochs. Based on empirical observations, the model typically converged and triggered the early stopping condition between 40 to 60 epochs. Furthermore, to prevent gradient explosion during sequence modeling, a strict Gradient Clipping strategy was applied, limiting the maximum gradient norm to 1.0 (Table 10).

#### Parameter configurations

**Table 10:** Summary of hyperparameter configurations for the proposed framework.

Module	Parameter	Value / Setting
DTW & K-Medoids	Window Size (*w*)	30
	Number of Clusters (*K*)	3
	Optimization Method	FasterPAM
	Maximum Iterations	300
VMD	Number of Modes (*K*)	5
	Bandwidth Penalty (*α*)	2000
	Noise Tolerance (*τ*)	0
	Convergence Tolerance	$1 \times 10^{-7}$
CEEMDAN	Ensemble Trials	50
	Noise Addition Strength (*ε*)	0.05
1DCNN	Kernel Size	3
	Dilation Rate	1 (Layer 1), 2 (Layer 2)
	Hidden Dimension	64
	Activation Function	ReLU
S-Mamba Block	Number of Layers	2
	Hidden Dimension	64
	Expansion Factor (*E*)	2 (Inner Dim = 128)
	Parameter Initialization	$\textbf{A}$: *łn (0.01)*, $\textbf{B}$ & $\textbf{C}$: $\mathcal {N}(0, 0.02^2)$
	Activation Function	SiLU (Swish)
	Normalization & Residual	LayerNorm, Pre-activation Residual
Training Setup	Look-back Window Size	30
	Prediction Horizon	3 (Direct Strategy)
	Optimizer	AdamW
	Learning Rate	0.001
	Batch Size	32
	Epochs	Max 100 (Early Stopping at *∼*40-60)
	Gradient Clipping Norm	1.0
	Loss Function	MSE

#### Experimental environment

To ensure fair comparison and experimental reproducibility, all models were trained and evaluated in the same computational environment. The specific hardware and software configurations of the deep learning server used in this study are detailed in Table 11.

**Table 11:** Hardware and software specifications of the experimental environment.

Category	Component	Specification
Hardware	CPU	Intel Xeon(R) Gold 6459C @ 3.00GHz (12 Cores)
	GPU	48 GB vGPU
	RAM (Memory)	96 GB
	Storage	30 GB System Disk + 50 GB Data Disk
Software	GPU Driver Version	595.58.03
	CUDA Support	*≤* 13.2
	Operating System	Linux (Ubuntu 20.04)
	Programming Language	Python 3.9
	Deep Learning Framework	PyTorch 2.0.1

#### Computational complexity and efficiency

To evaluate the model’s computational complexity, we tracked the parameter counts, training and inference times, and memory consumption. Since the proposed framework employs a weather-clustering strategy (DTW + K-Medoids), the overall predictive system consists of three weather-specific sub-models (Sunny, Cloudy, and Rainy). The total computational cost is calculated as the aggregation of these individual models.

As shown in Table 12, the aggregated system maintains a compact parameter size compared to standard large-scale baselines like Transformers, which contributes to its efficient training convergence and low-latency inference. Regarding memory usage, the peak VRAM consumption remains low during both individual model training and bulk inference on the largest test subset (Sunny).

Furthermore, the Mamba architecture utilized in our framework operates with a linear time complexity $\mathcal {O}(L),$ avoiding the $\mathcal {O}(L^2)$ computational bottleneck typical of standard self-attention mechanisms. The quantitative results in Table 12 objectively demonstrate the framework’s computational efficiency and its feasibility for practical deployment.

**Table 12:** Quantitative summary of the computational complexity, efficiency, and memory consumption of the proposed total framework.

Complexity metric	Quantitative value (total/aggregated)
Total Parameter Counts	197,961
Total Training Time	1633.25 seconds (*∼*27.2 mins)
Average Inference Time	0.0114 ms / sample
Peak Training Memory	64.38 MB (Maximum sequential peak)
Peak Inference Memory	2.91 GB (Bulk inference for 22,802 samples)
Computational Complexity (FLOPs scale)	$\mathcal {O}(L)$ Linear Time Complexity

## Data Availability

The photovoltaic power generation dataset used in this study was obtained from the Desert Knowledge Australia Solar Centre (DKASC), Alice Springs, Australia. The data is publicly available and can be freely downloaded from http://dkasolarcentre.com.au/download. This dataset includes historical PV power output, solar irradiance, temperature, and other meteorological variables from multiple PV systems. Detailed information on data selection, preprocessing, and the specific time period used is provided in the Methods section.

## Code availability

The custom code developed for this study, including the DTW-KMedoids clustering, VMD-CEEMDAN decomposition, and 1DCNN-SMamba prediction framework, is openly available in the GitHub repository at https://github.com/BaiKaiShui-Research/PV-Power-Forecasting-1DCNN-SMambaGitHub Repository. A sample dataset is also provided in the repository to demonstrate the full pipeline.
